# Organization of A^3^-coupling compounds by magnetic nanocomposite bimetallic in green conditions

**DOI:** 10.1371/journal.pone.0312758

**Published:** 2025-02-19

**Authors:** Mansour Binandeh, Mohammad Ali Nasseri, Ali Allahresani

**Affiliations:** Basic of Science, Chemistry Department, University of Birjand, Birjand, Iran; Beijing University of Chemical Technology, CHINA

## Abstract

The current project reports two chemical reactions of A^3^ coupling and creation of three reactans simultaneously and in domino form by bimetallic magnetic nanocatalyst (cobalt/manganese). This bimetallic nanocatalyst was heterogeneously synthesized by a simple chemical co-precipitation method and identified and analyzed by FE-SEM, TEM, VSM, ICP, BET, FT-IR, EDX, etc. analyses. The experimental results showed that the structure of the nanocatalyst is very regular and flexible, and its surface has spaces in nanometer sizes to carry out these chemical reactions. The efficiency and power of the nanocatalyst is very high and it has a special feature for easy separation from the reaction by an external field. The performed triple coupling reactions include the formation of the imide product, the propargylic product. The derivatives of this product each have a high percentage of 76% and selectivity of 98%. These products are widely used in new precursors for making antioxidant and antimicrobial drugs in medical and chemical industries.

## Introduction

Catalysts used in chemical and medical industries have become very practical nanocatalysts in nanometer dimensions and their nanotechnological synthesis has attracted the attention of many researchers. The used nanocatalysts have different types, among them, magnetic nanocatalysts have wider applications such as; Coupling, synthetic and multicomponent reactions [1]. Therefore, in the nanotechnology industry, the creation of dimensions and particles in the nanometer range has been developed and special attention has been paid to the regular and crystalline structures of nanoparticles in order to evaluate the catalytic power of nanocatalysts [2]. These nanoparticles have been very useful and controlled, among which, magnetic nanoparticles have recently found wide applications. Among their applications in the medical industry, we can mention the absorption and purification of bio-medical molecules [3].

### Magnetic nanoparticles

A very important issue regarding these nanocatalysts is the influence of their surface in the vicinity of air, which is susceptible to oxidation. Therefore, they reduce the performance of nanocatalysts and cause their nanoparticles to clump, which is the solution to prevent this by using bio-organic coatings. One of these coatings is silica nanoparticles, which have antibacterial properties and turn magnetic nanoparticles into biodegradable and medically active nanocatalysts, which are used in the stabilization and absorption of drug biomolecules, targeted drug delivery, and antibacterial properties [1, 2].

Another reason for the use of heterogeneous catalysts is due to the easy extraction from the reaction medium and also the possibility of using them several times due to their high performance, which has been considered by most researchers [4]. At the same time, the use of these heterogeneous catalysts at the nanoscale has been optimally used due to their large surface area to perform different chemical reactions and also the possibility of loading different ligands by migrating different metals on nanoparticle substrates [5, 6].

### Schiff’s base structures, organic linkers and bimetallic nanoparticles

3-Chloroisopropyl triethoxysilane organic linker, in addition to being affordable, has a chlorine leaving group to establish a connection between the silica coating and the organic linker with the nucleophilic group. The organic linker of Lysine is one of the structural proteins in the body of living organisms, which is in the form of an alpha carboxylic amide compound. This organic compound forms a cluster structure and twists called Schiff-Base structure (by creating an amide bond). The end band of the Lysine compound has a carboxyl group with high and strong resonance, which reduces the possibility of connecting to another organic group. For this purpose, tosyl structures are used. After creating the leaving group on the Lysine substrate, the next organic linker can be added to create a robust structure of the magnetic nanocatalyst. Morpholine, one of the compounds with nerve control power in the nerve receptors of living organisms, is a suitable option for connecting to the carbonyl bond of Lysine and creating an amide bond. In the structure of the catalyst, at the stage when the Lysine structure in the reaction medium is converted to the ethanol solvent as an acid-base ion pair, its pH is different on both sides of the molecule and finally on the acidic (carboxylic) side. On other hand, it is a Schiff-base structure that can be used as a green catalyst in organic reactions [5–9].

Nowadays, to carry out chemical reactions, a nanometal in the structure of magnetic nanocatalysts is needed to speed up the process. Palladium metal is one of the most widely used and high-performance metals used by many researchers, but its high price, the saturation of its pores every time it is reused, and its hard regeneration have made researchers prefer other nanoparticles. Cobalt and manganese nanoparticles have high efficiency compared to palladium metal due to high leaching, low price, availability, ease of working with them, and the efficiency of products in every chemical reaction is much better and high [10–16].

### A^3^-coupling reaction

One of the most important issues from the point of view of scientists is related to pair reactions, which were first named by prominent scientists such as Suzuki, Heck, Buchwald and Sonogashira. The results of research on the use of different nanocatalysts in these reactions were widely reviewed [17–23]. One of the most recent emerging cross-couplings is related to the three-component coupling, which is the result of sp^2^, sp carbons and N (of morpholine) for A^3^-coupling, which is co-linked to A^3^ propargylic compounds, and finally a new chiral compounds is occurred. The obtained products have valuable carbon and nitrogen groups, which are of particular importance in the medical-genetic industry for the production of various protein and lycoprotein vitamins and the modification of chemical drugs [24].

In this part, two important issues are raised, one is related to the synthesis of A^3^ compounds and the second one is the synthesis of propargylic compounds. As we know, The A^3^ coupling (also known as A^3^ coupling reaction or the aldehyde-alkyne-amine reaction), coined by Prof. Chao-Jun Li of McGill University, is a type of multicomponent reaction involving an aldehyde, an alkyne and an amine which react to give a propargylamine. The reaction is carried out through the process of removing a water molecule [25] and requires a metal catalyst that is usually based on ruthenium/copper, gold or silver [25]. A chiral catalyst can be used to create a selective reaction and produce a chiral amine. The solvent can be water [26]. In the catalytic cycle, the metal activates the alkyne to a metal acetylide, the amine and aldehyde combine to form an imine, which then reacts with the acetylide in a nucleophilic addition [27]. The type of reaction was reported independently by three research groups in 2001–2002 [28–30], a report on a similar reaction dates back to 1953 [31, 32]. If the amine substituents have an alpha hydrogen, and provided a suitable zinc or copper catalyst is used, the A^3^ coupling product may undergo an internal hydride transfer and further fragmentation to form an allene in the Krabe reaction. In 2018, Mr. Kaur and co-workers reported the coupling compounds A^3^ by copper nanocatalyst with 78% efficiency [33]. In 2019, Mr. Jessin was able to perform a complete review of A^3^ coupling compounds [34], in 2018 [35–37], in 2020 [38, 39], in 2022 [40], and in 2023 [41, 42] also reported.

Propargylic compounds years ago in the form of molecular formula C_3_H_3_NO with a molecular weight of 69.06 g/mol, propargylic is a doubly unsaturated 5-membered ring having one oxygen atom at position 1 and a nitrogen at position 3 separated by a carbon in-between. It was first prepared in 1947, has a boiling point of 69°C and is a stable liquid at room temperature [43]. In 2013, Mr. Srivastava and his colleagues reported derivatives of compounds with aldo-pyrdino-acetyrene by CuFe_2_O_4_ nanoparticles. Although these synthesized compounds had an efficiency of 88% [44]. In 2020, Mr. Mariconda and his colleagues reported the synthesis of propargylic-like compounds by Au/Ag@Fe_3_O_4_ nanocatalyst and the efficiency was 89% [45], and others [46–52].

**Fig 1:**
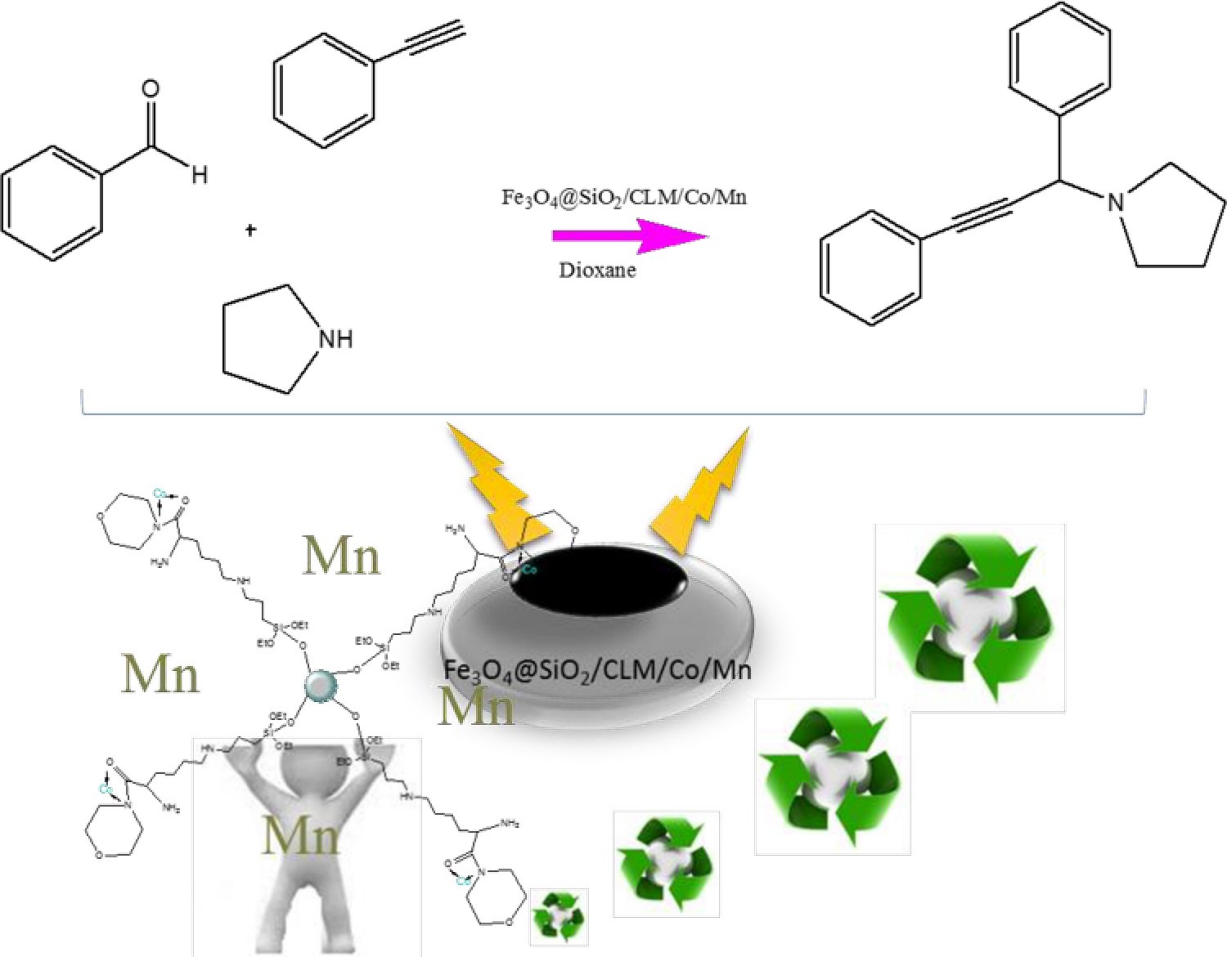
A^3^ coupling reaction. General of A^3^-coupling reaction with Fe_3_O_4_@SiO_2_/CLM/Co/Mn magnetic nanocatalyst.

In this project, magnetic nanocatalysts containing two metals manganese/cobalt have been used in an ideal condition for the synthesis of propargylic compounds. As can be seen below, a new reagent has been used for the synthesis of these compounds, which is a two-step reaction. These new compounds as new chemical drugs can be used in the pharmaceutical-medical industry as oxidant and antimicrobial drugs (Fig 1).

## Materials and methods

All reagents used meet the standards and the solvent used is deionized water of 18MO/cm. Lysine, C_6_H_14_N_2_O_2_ (molecular weight 146.19 g/mol, 99.9 99% purity), Morpholine, C_4_H_5_NO (with a molecular weight of 111.10 g/mole, 99.9 99% purity), (3-chloroiso-propyl) triethoxysilane or CPTES, (molecular weight 198.72, 97% purity), Manganese (II) acetate (Mn(OAc)_2_, molecular weight 173.0273 g/mol, 99% purity), C_4_H_6_CoO_4_, (Cobalt (II) acetate, molecular weight 177.02124 g/mol, 99% purity) of Sigma Aldrich. FeCl_2_.4H_2_O, Fe(Cl)_3_.6H_2_O, deionized water, argon gas, NaOH (34% aqueous solution), TEOS, HCl, dioxane and methanol was purchased from Sino- pharm Chemical Reagent Co. (Shanghai, China).

Powder XRD of the prepared catalyst was performed using a Philips PW 1830 X-ray diffractometer with a Co Kα source (λ = 1.788965 Å) in the Bragg angle range 10–80° at 25°C. FT-IR spectra were obtained using a FT-IR spectrometer (Vector 22, Bruker) in the range 400–4,000 cm^−1^ at room temperature. SEM analysis was conducted using a VEGA//TESCAN KYKY-EM 3200 microscope (acceleration voltage of 26 kV). TEM experiments were conducted with a Philips EM 208 electron microscope. EDX analysis of the catalyst was conducted with a VEGA3 XUM/TESCAN. TGA was performed with a Stanton Red Craft STA-780 (London, UK). NMR spectra were performed with a Bruker DRX-400 advancing instrument (300.1 MHz for ^1^H, 75.4 MHz for ^13^C). The spectra were obtained with samples in CHCl_3_-d1 as a solvent. Magnetic measurements were carried out using a VSM instrument (MDK, model 7400). Melting points were evaluated with an Electrothermal 9100 apparatus.

### Nanoparticle synthesis method

One of the methods of synthesis of magnetic nanoparticles is the chemical Co-precipitation method [1, 2], which is the best method due to its ease and creation of a high percentage of synthetic products. The work is based on the creation of a heterogeneous catalyst that is in the aqueous phase and in the organic phase (where the reactants are), phase transfer occurs and the reaction is initiated by the catalyst. This method is for making magnetic nanoparticles in the form of iron oxide, which is obtained by combining two iron salts, Fe (II) (FeCl_2_.4H_2_O) and Fe (III) (FeCl_3_.6H_2_O) with a ratio of 1: 2.

### Synthesis of Fe_3_O_4_ magnetic nanoparticles

**Fig 2:**
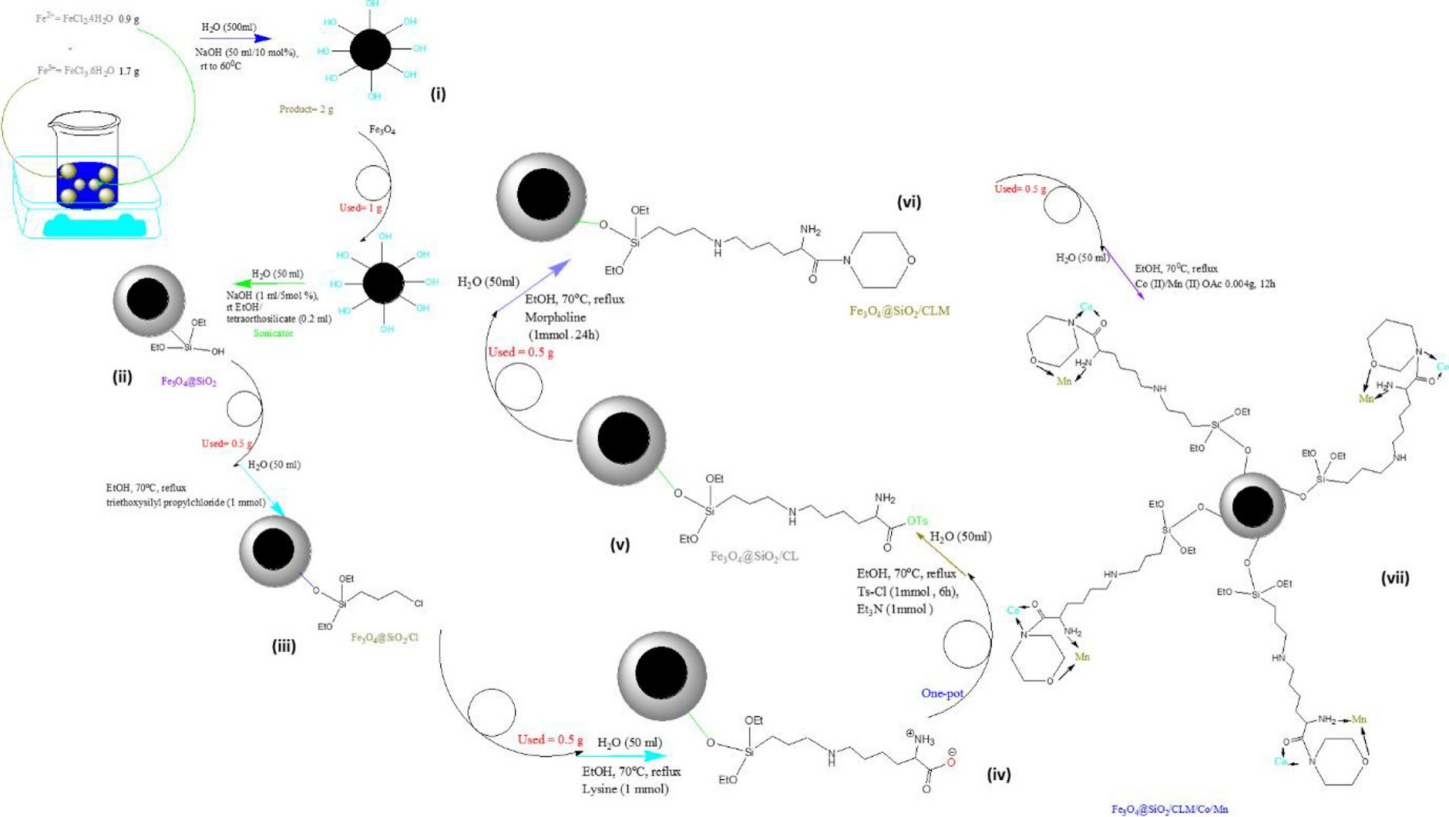
Steps the synthesis of Fe_3_O_4_@SiO_2_/CLM/Co/Mn magnetic nanocatalyst. 2-i) Fe_3_O_4_, 2-ii) Fe_3_O_4_@SiO_2_, 2-iii) Fe_3_O_4_@ SiO_2_/Cl, 2-iv) Fe_3_O_4_@ SiO_2_/CL, 2-v) Fe_3_O_4_@ SiO_2_/CLOTs, 2-vi) Fe_3_O_4_@ SiO_2_/CLM and 2-vii) Fe_3_O_4_@ SiO_2_/CLM/Co/Mn.

The synthesis of magnetite magnetic nanoparticles is based on the core/shell structure, which is carried out through a series of steps. To make its core, iron oxide consisting of two salts of Fe (II) (0.9 g, 0.2 mol% FeCl_2_.4H_2_O), Fe (III) (1.7 g, 0.5 mol% FeCl_3_.6H_2_O) is used in 300 ml of distilled water. This reaction is carried out at room temperature (25°C) for 2 hours (h). After 2 h, the reaction slowly increased to 65°C. As soon as this temperature is reached, 5 ml of 10% molar sodium hydroxide solution is regularly added to the reaction solution. The reaction continues for another two hours in the same way at a temperature of 65°C at the average speed of the magnet. Finally, the obtained product is magnetite (Fe_3_O_4_) magnetic nanoparticles (which are the core of the catalyst). Then the product is separated from the reaction solution by an external magnetic field, placed on a heater in the room (25°C) and washed with 50°C of distilled water and again (using a magnet) the product is separated from the reaction. This is done 3 times and washed with 20 ml of Merck ethanol, and this time the product is separated by a magnet and transferred to an oven under a temperature of 60°C for 6 h. At this temperature, the product is completely dried and loses its surface water. Then the product is transferred to a vacuum oven under 55°C for 12 h to lose its internal water. The reason for these two models of magnetic nanocomposite drying is the presence of water, which, with its hydroxyl groups, causes interference in the chemical location of the amine group in IR analysis, so it dries to obtain clear and regular peaks. Finally, the obtained product is collected in a yellowish brown color (Fig 2–i).

### Nanoparticle core / shell structure with metal coating

After the synthesis of magnetic nanoparticles, its catalytic surface should be covered by silicate nanoparticles. Therefore, for this purpose, first, 0.5 g of the synthesized catalyst was dispersed in 50 ml of distilled water in a weighed container and placed on the sonicator for 1.5 hours. Then, in a separate reaction container, 4 ml of tetraorthosilicate was poured on 5 ml of ethanol and added to the previous solution. During this reaction, 0.5 ml of 10% sodium hydroxide solution should be added to the reaction solution and the reaction should continue in the sonication stage for 2 hours. Then it continues for another 2.5 h in the heater at room temperature i.e. 25°C and finally the core/shell product containing silica nanoparticles coated on the surface of the magnetite nanocatalyst was synthesized (Fe_3_O_4_@SiO_2_) (Fig 2–ii). The obtained nanocatalyst was washed, dried, collected and weighed according to the above method. To add each of the ligands, the reaction is carried out one by one, so that the first 0.5 g of (Fe_3_O_4_@SiO_2_) nanocatalyst is dissolved in 50 ml of distilled water and 1 mmol of triethoxysilyl propyl chloride. Then 25 ml of ethanol was added to the previous solution and the whole solution was refluxed at 70°C for 24 hours. After one day, the product is washed several times with ethanol and distilled water and dried in an oven at 65°C and collected (Fe_3_O_4_@SiO_2_/Cl) (Fig 2–iii). In the next steps, two linkers and organic ligands are placed on the chlorinated magnetic nanocatalyst substrate in such a way that, first, 0.5 g of magnetic nanocatalyst was dispersed in 50 ml of deionized water. In another container, 1 mmol of organic lysine linker was dissolved in 20 ml of Merck ethanol and added to the reaction container. The reaction mixture was refluxed at 70°C for 24 h. Then, the magnetic nanocatalyst (Fe_3_O_4_@SiO_2_/CL) was washed by Merck ethanol/double ionized water, dried and weighed. The reaction product was a mixture of neutral lysine and charged lysine (Fig 2–iv, 2-v). In the next step, about 0.5 g of this nanocatalyst was dispersed in 50 ml of distilled water and reacted in the presence of 1 mmol of tosyl chloride and 1 mmol of triethylamine (until the oxytosyl group, which is a suitable leaver). In another container, 1 mmol of morpholine organic ligand was dissolved in 20 ml of Merck ethanol and added to the reaction container. The reaction was refluxed at 70°C for 24 h. Finally, the amide reaction product from the magnetic nanocatalyst (Fe_3_O_4_@SiO_2_/CLM) was washed several times with Merck ethanol/double ionized distilled water, dried and weighed (Fig 2–vi). In the final step, about 0.5 g of magnetic nanocatalyst (Fe_3_O_4_@SiO_2_/CLM) was dispersed in 25 ml of distilled water. In another side, 0.004 g of manganese (acetate)_2_ and cobalt (acetate)_2_ metal salts were dissolved in 25 ml of Merck ethanol and added to the reaction vessel. The reaction was refluxed for 12 h under a temperature of 70°C. Finally, the obtained magnetic nanocatalyst (Fe_3_O_4_@SiO_2_/CLM/Co/Mn) was washed by double ionized water and Merck ethanol several times, dried and collected. The final product was a yellowish brown magnetic nanocatalyst (Fig 2–vii).

## Results and discussion

First, the synthesized nanocatalyst must be analyzed to fully confirm its structure. For this purpose, a series of analyses such as SEM, TEM, EDX, VSM and TGA have been used. The main purpose of these analyses is to study their crystalline and core / shell structure (magnetic nanocatalyst) and to regulate its particle size in the range of 50–100 nm. In the following, each of the mentioned analyses will be discussed.

### Analyses tools of detection

#### SEM, TEM and the elemental scanning analyses

**Fig 3:**
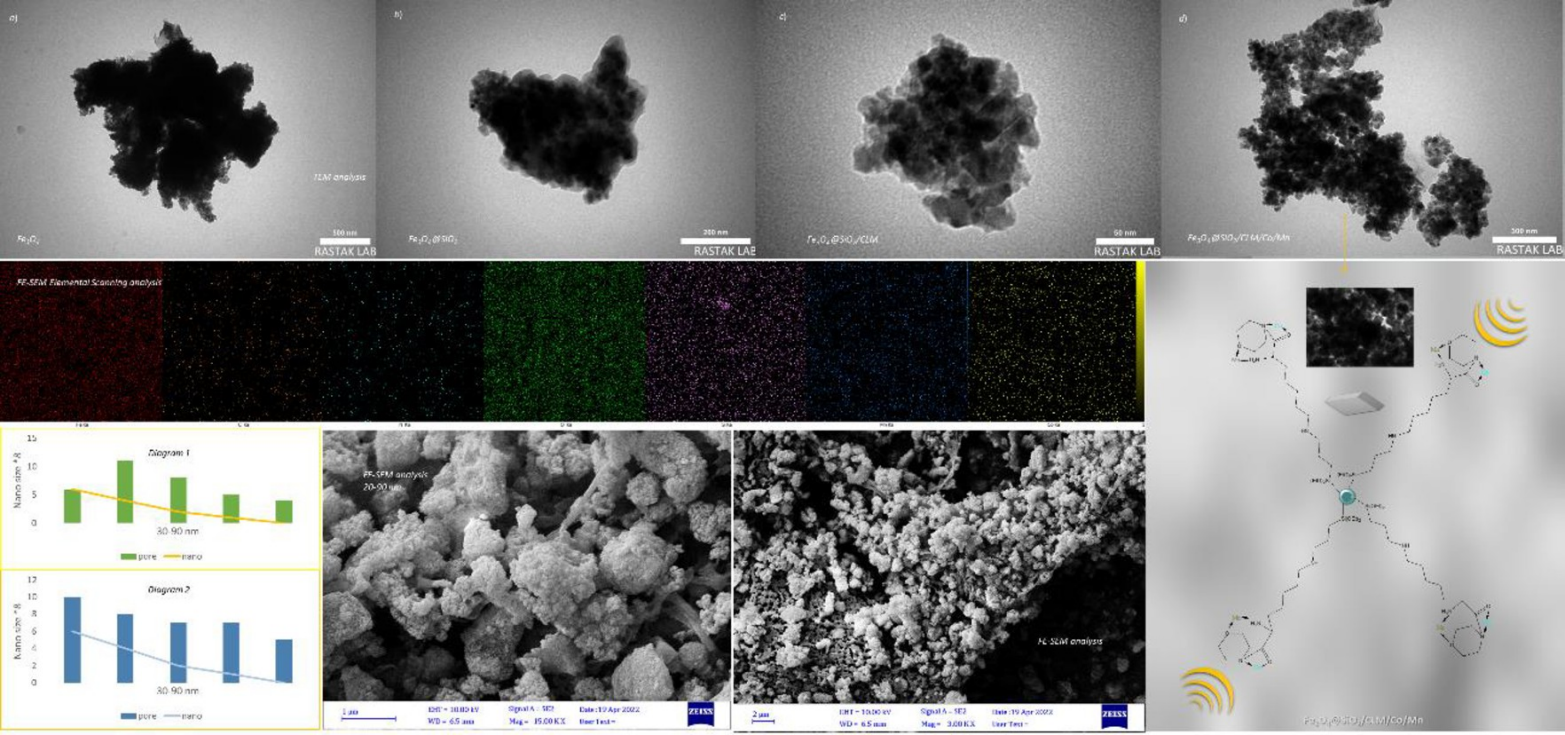
TEM, SEM and the Elemental Mapping analyses with a graphs that represents the order of nanoparticles. a) Fe_3_O_4_, b) Fe_3_O_4_@SiO_2_, c) Fe_3_O_4_@SiO_2_/CLM and d) Fe_3_O_4_@SiO_2_/CLM/Co/Mn (TEM analysis), Fe, C, N, O, Si, Mn, Co (Elemental Scanning analysis), FE-SEM (left and right) and Diagrams 1, 2.

One of the analyses that showed the surface structure of nanoparticles in clear images is SEM and TEM analysis. In these analyses, images of the morphology of the core/shell structure of nanoparticles and to some extent the size of their particles were taken with more zoom to investigate the structure of nanoparticles. Therefore, very useful information was obtained about the crystalline composition of nanocatalysts on the scale of nanometers and even micrometers, which shows the extent of the surface of nanocatalysts. The results of investigations show that the size of magnetic nanoparticles is between 30–90 nm and in the range of 80 nm. The elemental mapping analysis also showed images of the distribution of iron, silicon, manganese and cobalt particles on the magnetic nanocomposite substrate, and the particles are very regular and uniform and almost the same size in the nanocomposite structure. Also, in these analyses, the crystalline structure of the magnetic nanocatalyst obtained from silica coating, isopropyl triethoxysilyl chloride coating, Lysine, morpholine ligands and finally the loading of manganese/cobalt metals have received much attention (Fig 3, Diagrams 1, 2).

#### The FTIR analysis

**Fig 4:**
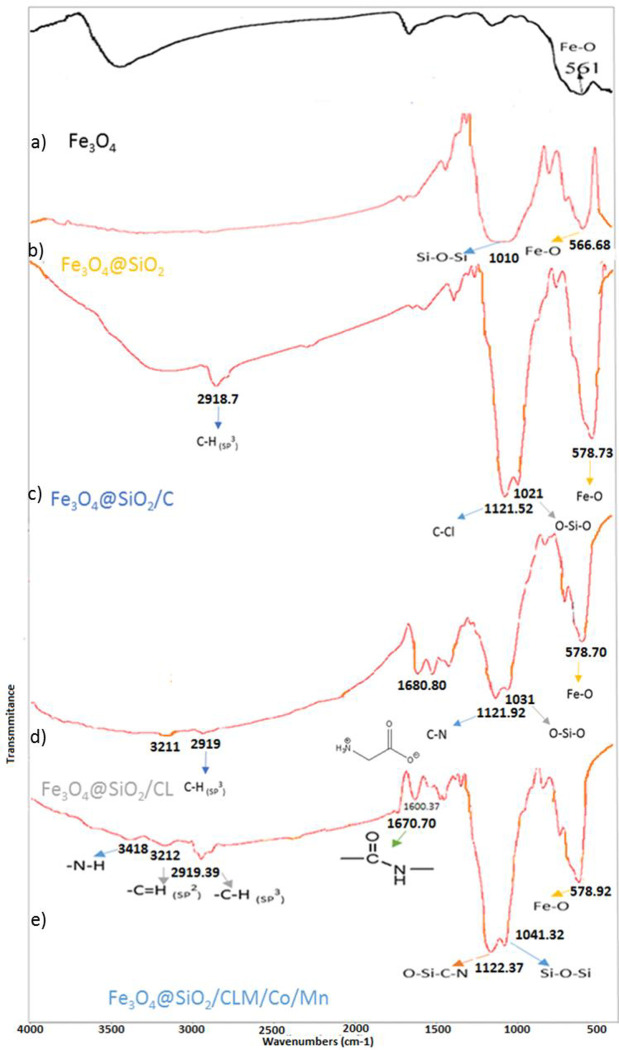
FT-IR analysis Fe_3_O_4_@SiO_2_/CLM/Co/Mn. 4a) Fe_3_O_4_, 4b) Fe_3_O_4_@SiO_2_, 4c) Fe_3_O_4_@SiO_2_/Cl, 4d) Fe_3_O_4_@SiO_2_/CL and 4e) Fe_3_O_4_@SiO_2_/CLM/Co/Mn.

The FTIR analysis is one of the most important analyses for identifying functional groups in a combination, which shows which functional groups exist and what links are formed between them. This analysis is reported on the basis of data per centimeter and analyses between 400–4000 cm^-1^. In the nanocatalyst synthesized in this project, the iron-oxygen bond in the range of 500 cm^-1^ shows the structure of its core, ie magnetic nanoparticles, which is a very large peak that gradually becomes more regular and sharper as the nanocatalyst coats and ligandes. This indicates the strength of the building of the structure of the catalyst core has been identified, it is the turn of the loose structure or shell, which consists of a silicate in the form of a silyl-oxygen-silyl bond that peaks in the range of 1000 cm^-1^ [53], which is quite large in the latter form, strong shows the presence of silica coating prevents excessive oxidation of the nanocatalyst surface and gives it good biocompatibility and biodegradability. The first ligand to be placed on a silica coating was triethoxy propyl chloride, which gave exactly the bulge next to the silicate peak above its peak, which is clearly a bifurcated peak at the bottom (>1100 cm^-1^). The other ligand, Lysine, attacks the previous ligand with its free nitrogen and releases chlorine, producing a hydrochloric acid that is removed from the reaction medium by heat and solvent. A covalent bond is then formed between Lysine nitrogen and the previous carbon ligand, which peaks at 1121 cm^-1^ (strength -C-N) and 1400 cm^-1^ (bending -C-N). The other free part of Lysine is ethanol-soluble in the form of an alpha-amino acid compound that gives a weak peak in the region of 1680 cm^-1^ due to the high resonance between the nitrogen and the carboxyl group. In another form, a covalent bond is formed between the nitrogen of the Morpholine ligand and the carboxylic moiety of Lysine, which is an amide bond that peaks at 1600 cm^-1^ due to the high resonance between the two adjacent nitrogen groups and the ketone group. If we compare the shapes, we come to the conclusion that the peaks have become more regular and the structure of the nanocatalyst has become more completed is related to carbon-hydrogen (C-H SP^3^ = >2990 cm^-1^, C-H SP^2^ = >3180 cm^-1^), carbon-dual carbon (C-C SP^3^ = 1000–1200 cm^-1^, C = C SP^2^ = 1400–1600 cm^-1^), carbon-nitrogen (C-N SP^3^ = 1200–1400 cm^-1^), carbon-oxygen (C-O = 1000–1200 cm^-1^, -C = O carboxylic acid1680 cm^-1^, -C = O amide = 1600 cm^-1^) and free amine bonds (3418 cm^-1^) (Fig 4A–4E).

#### EDX analysis

**Fig 5:**
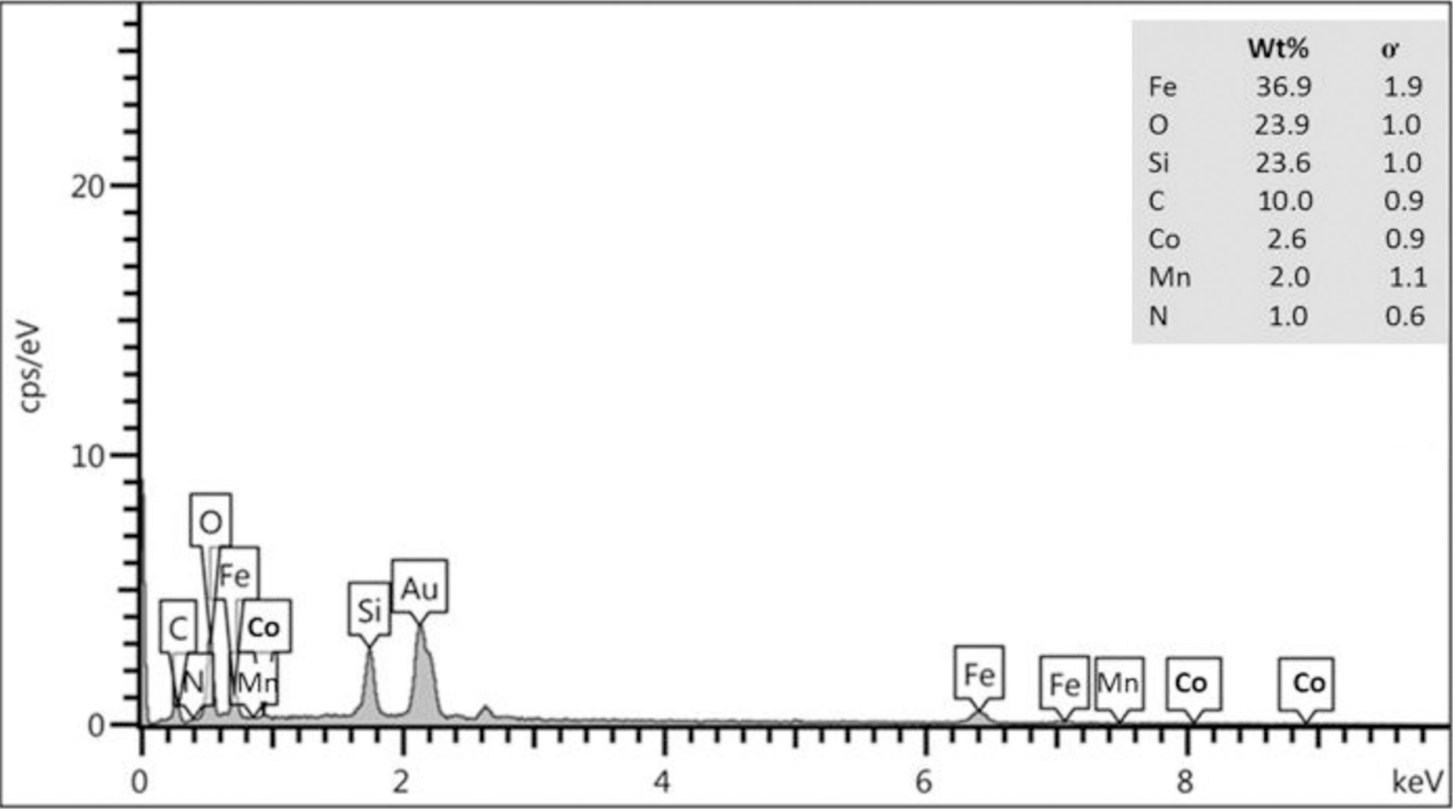
EDX analysis of Fe_3_O_4_@SiO_2_/CLM/Co/Mn nanocatalyst. Wt % measuring of Fe, O, Si, C, N, Co and Mn.

EDX analysis can be a valuable tool for identifying bonds between elements. The percentage of an element is combined with the KeV unit, which is not a numerical unit and is only expressed as a mass percentage (Wt %) (Fig 5). For the Fe_3_O_4_@SiO_2_/CLM/Co/Mn magnetic nanocatalyst, it proves the existence of elements such as (Fe, O, Si, N, C respectively, 36.9, 23.9, 23.6, 1, 10%). The mass percentage results for two metals, Co and Mn, 2.6% and 2%, respectively, show that the catalytic power at an efficiency of over 90% is more than the basic state.

#### The XRD spectrum of Fe_3_O_4_@SiO_2_/CLM/Co/Mn

**Fig 6:**
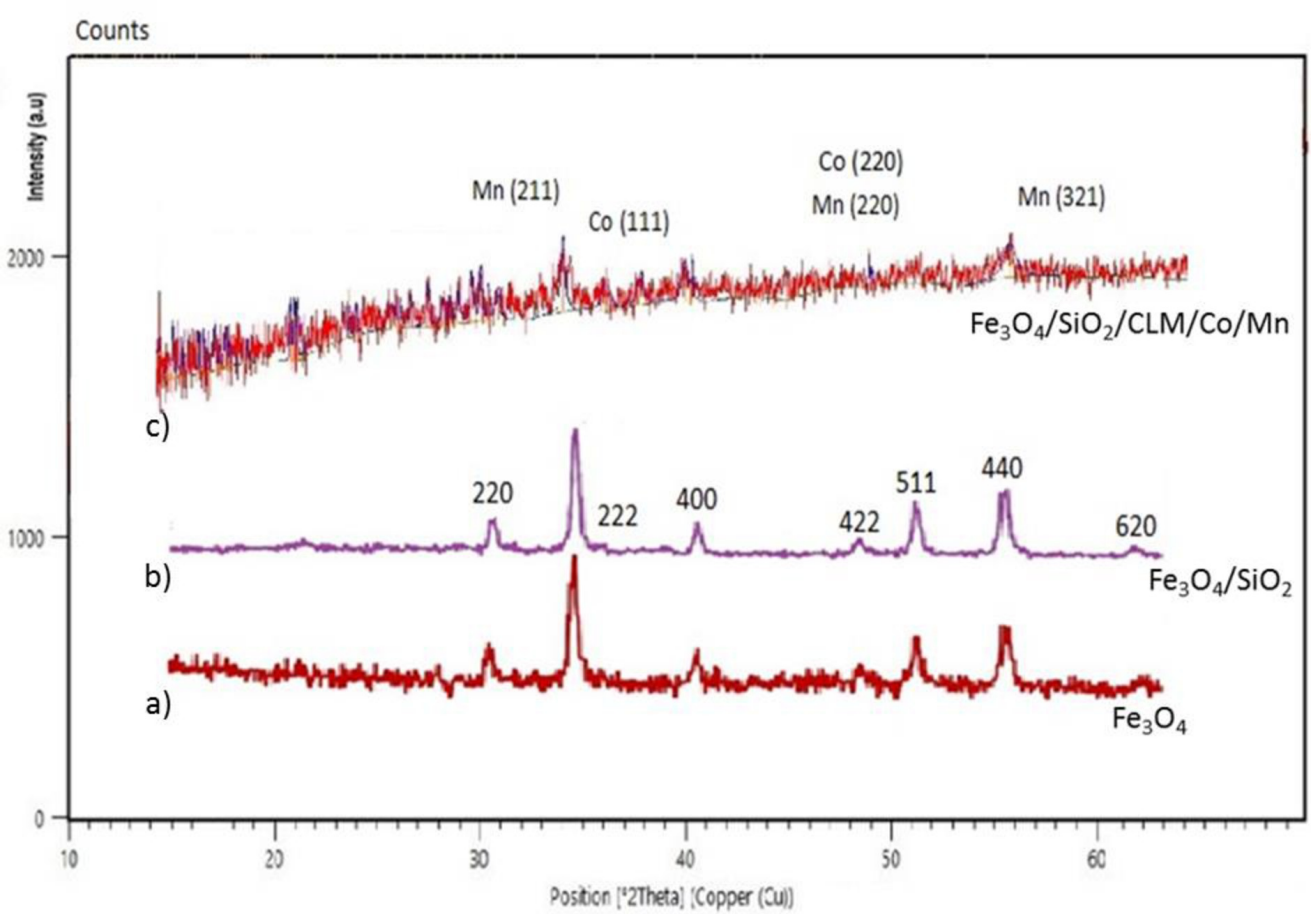
XRD analysis to detect the structure of magnetic nanocatalyst. 6a) Fe_3_O_4_, 6b) Fe_3_O_4_@SiO_2_ and 6c) Fe_3_O_4_@SiO_2_/CLM/Co/Mn.

In another analysis, the structure of the nanocatalyst was analyzed by XRD analysis, X-ray diffraction, which shows energy levels in orbital layers and electron capacities based on electron transfer using X-rays. For this purpose, the XRD pattern of three broad peaks of the sample of Fe_3_O_4_ at diffraction angles (equivalent to 2 theta pixels) 30.1° and 35.4°, 43.2°, 53.7°, 56.9°, 62.9° in the lattice reflections from the (220), (311), (422), (440), (511) (Fig 6A). The results shown in Fig 6 (the first line of the graph) are confirmed based on the previous articles [54] that were synthesized in 2022–2023, the structure of Fe_3_O_4_. In the second line, which is covered with silica, the same numbers as before appear and the peaks are slightly sharper (Fig 6B). In the third and final lines, with the placement of organic linkers and manganese and cobalt metals, a series of new peaks have been created and previous peaks have been strengthened. Two new peaks in 2 thetas at 44 and 56°, three other peaks at 36, 56 and 63°, which are similar to the peaks at (111, 220), (211, 220, 321), which are related to the complexation of cobalt and manganese metals on the magnetic nanocomposite substrate, respectively (Fig 6C). An average crystallite size D  =  42 nm is calculated using the peak broadening in the Scherrer`s relation as described elsewhere [55, 56]. According to the investigations, the completed structure of the magnetic nanocatalyst has regular crystallography and heterogeneous surface, the size of the holes created by the two cobalt/manganese metals is about 25 nm. Also, two metals cobalt in oxidation states (II, III) and manganese in oxidation states (II, III, IV, V) gave a multicomponent structure to the magnetic nanocatalyst, which is a confirmation of its crystal structure.

#### UV analysis

**Fig 7:**
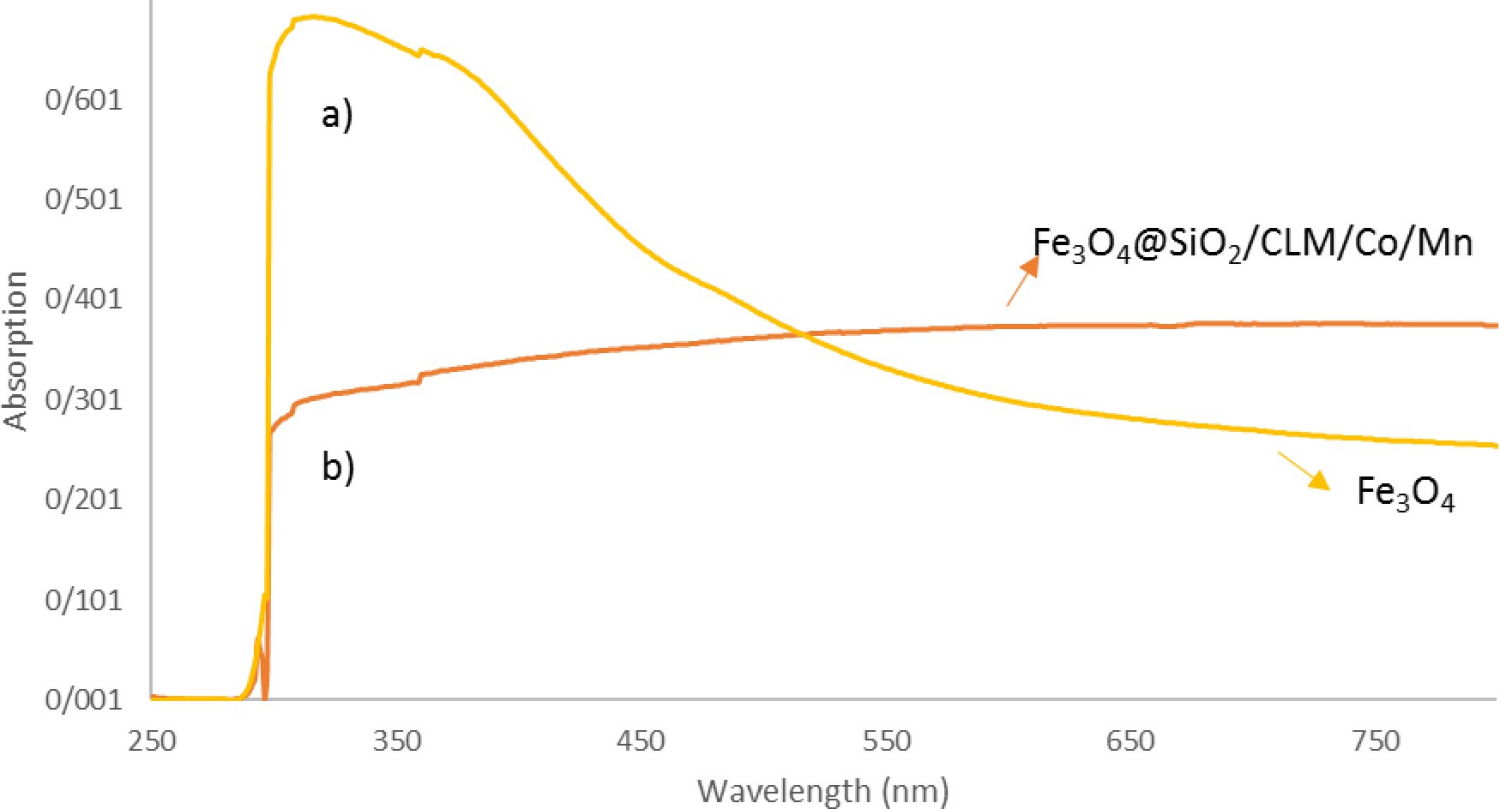
UV analysis by the data`s adsorption of completed nanocatalyst. 7a) Fe_3_O_4_ and 7b) Fe_3_O_4_@SiO_2_/CLM/Co/Mn.

In order to further investigate the structure of magnetic nanocomposite, another analysis called UV was used, which is based on the absorption parameter in the wavelength range of 400–600 nm. The placement of silica coating, linkers and organic ligands and manganese/cobalt metals on the magnetite magnetic nanocatalyst substrate was investigated. The results for magnetite nanoparticles showed a very high absorption (ʎ_max_ = 0.6), which can be seen in Fig 7A. At the wavelength of 400–600 nm, the absorption rate (ʎ_max_ = 0.12) of the completed nanocatalyst, with each of the silica coatings, organic binders and cobalt/manganese metals, has been significantly reduced (Fig 7B). According to these results, it seems that the structure of the magnetic nanocatalyst is complete and covered by linkers, organic ligands, and cobalt/manganese metals (II).

#### VSM analysis

**Fig 8:**
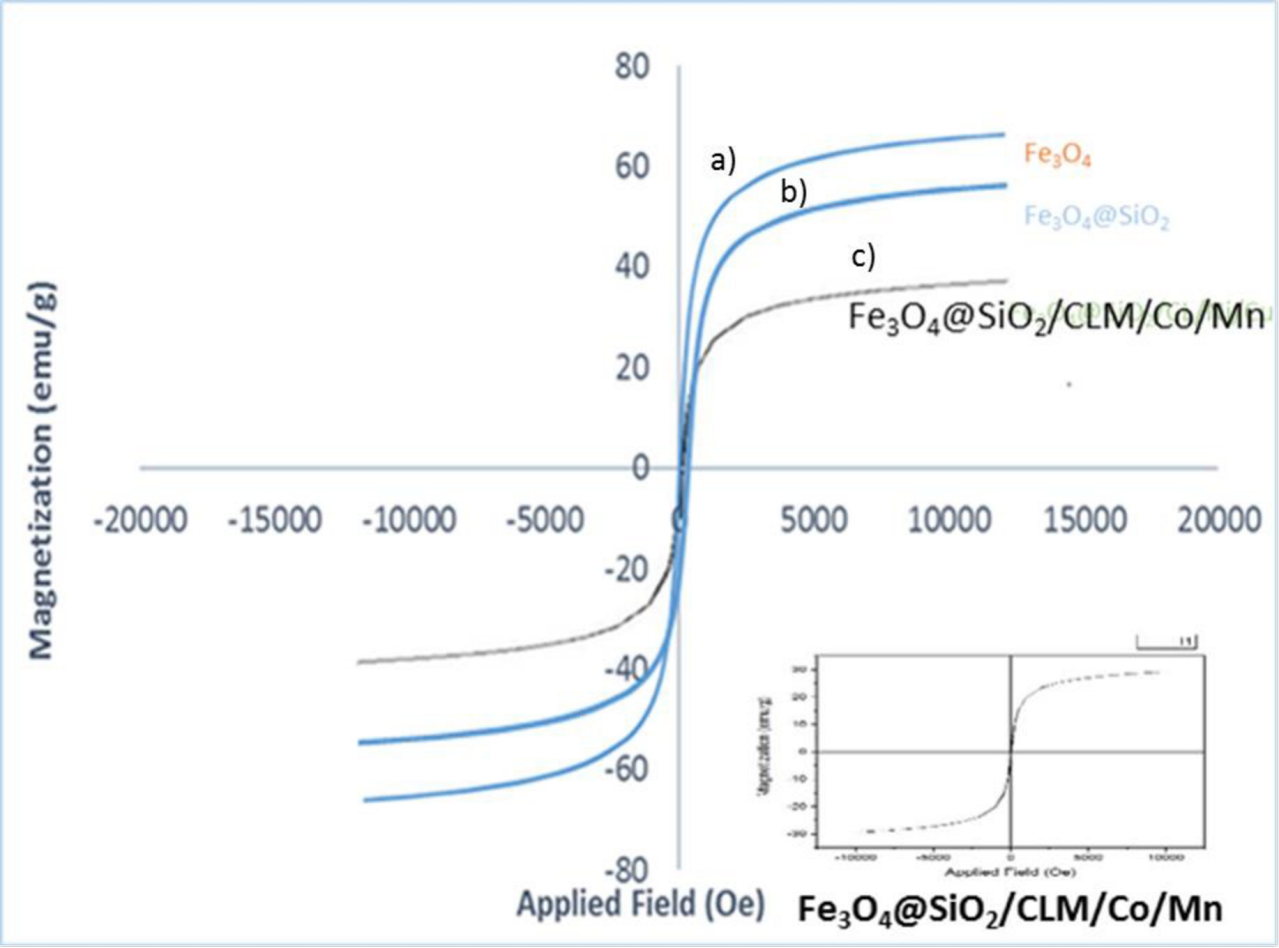
VSM analysis of magnetic properties of magnetic nanocatalyst. 8a) Fe_3_O_4_, 8b) Fe_3_O_4_@SiO_2_ and 8c) Fe_3_O_4_@SiO_2_/CLM/Co/Mn.

This analysis is used to measure the amount of magnetometer saturated based on the type of magnetic nanoparticles (para, dia and ferromagnetic) and the amount of magnetism in the magnetic field. Therefore, for this purpose, this analysis was used based on data suffering with criterion (emu/g) in the unit of magnetic field (kOe). Data for magnetic nanoparticles (Fe_3_O_4_) is made about 57 emu/g (Fig 8A), 25–100°C and PH = 8–12, for silica (Fig 8B)/ Lysine groups about 19, and 11 emu/g for Co/Mn fixed on magnetic nanoparticles, which after synthesis of it (Fe_3_O_4_@SiO_2_/CLM/Co/Mn) nanocomposite, the degree of magnetization shown is 34 emu/g (Fig 8C), that these are at a temperature of 25°C and at a temperature above 100°C, about 25% of these values are reduced, and finally this magnetic nanocatalyst retains more than 3/4 of its weight at this temperature, which is more than 27% higher than the other Nanocatalysts are stable. This decrease is due to the conversion of magnetic nanoparticles to superparamagnets, which are much more efficient than other nanocatalysts for A^3^-coupling reactions. The values in the diagram show that the magnetic rate of magnetic nanoparticles is very standard and of excellent quality compared to others. The products of coupling reactions by this nanocatalyst have about 25% more quality and adsorption properties than other nanocatalysts and are finally selected as a high efficiency nanocatalyst (Fig 8). The rate of decrease in magnetization and the increase in superparamagnetic properties depend on the overall structure of the magnetic nanocatalyst (including the type of ligands and the number of metals loaded). Due to the 35% reduction in magnetization in the structure of the magnetite (without ligand and metal loaded on its surface) compared to the complete structure of the nanocatalyst synthesized here, the catalytic and magnetic strength had a 90% effect on the reaction rate (magnetite itself It has 20% of catalytic power, which is 11% for each loaded ligand and 35% of metals in total).

#### TGA analysis

**Fig 9:**
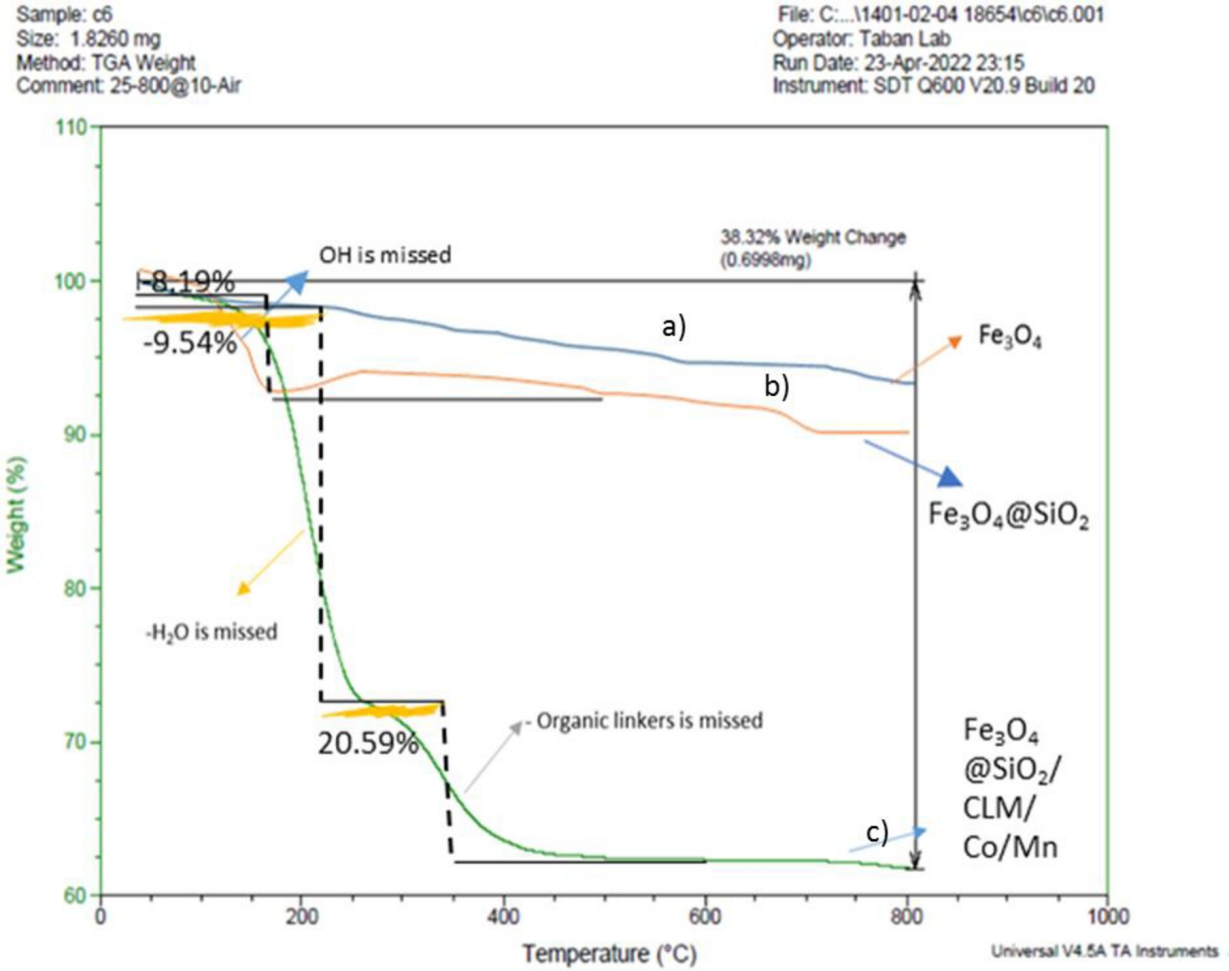
TGA analysis of nanocatalyst by steps. 9a) Fe_3_O_4_, 9b) Fe_3_O_4_@SiO_2_ and 9c) Fe_3_O_4_@SiO_2_/CLM/Co/Mn.

One of the important analyzes for further confirmation of the catalyst was thermogravimetric analysis (TGA). After further investigation in this analysis, in the temperature range of 0–800°C, the new magnetic nanocatalyst has shown good resistance up to 250°C (Fig 9A and 9B). The weight loss occurred in the temperature range of 250 to 400°C related to the loss of water from the inner and outer layers (which means that as the magnetic nanocatalyst dissolves in the water solvent, it must lose its excess water, so that the peaks related to hydroxide bands are removed in the IR analysis so that the obtained peak is sharp and free of hydrogen bonds with water molecules), and also the bending peak was related to the decay of organic ligands from the magnetic nanocatalyst structure (Fig 9C). The amount of mass reduction is about 62%, and what remains of the nanocatalyst structure is about 38% of the total mass (Fig 9).

#### BET and ICP analyses

**Fig 10:**
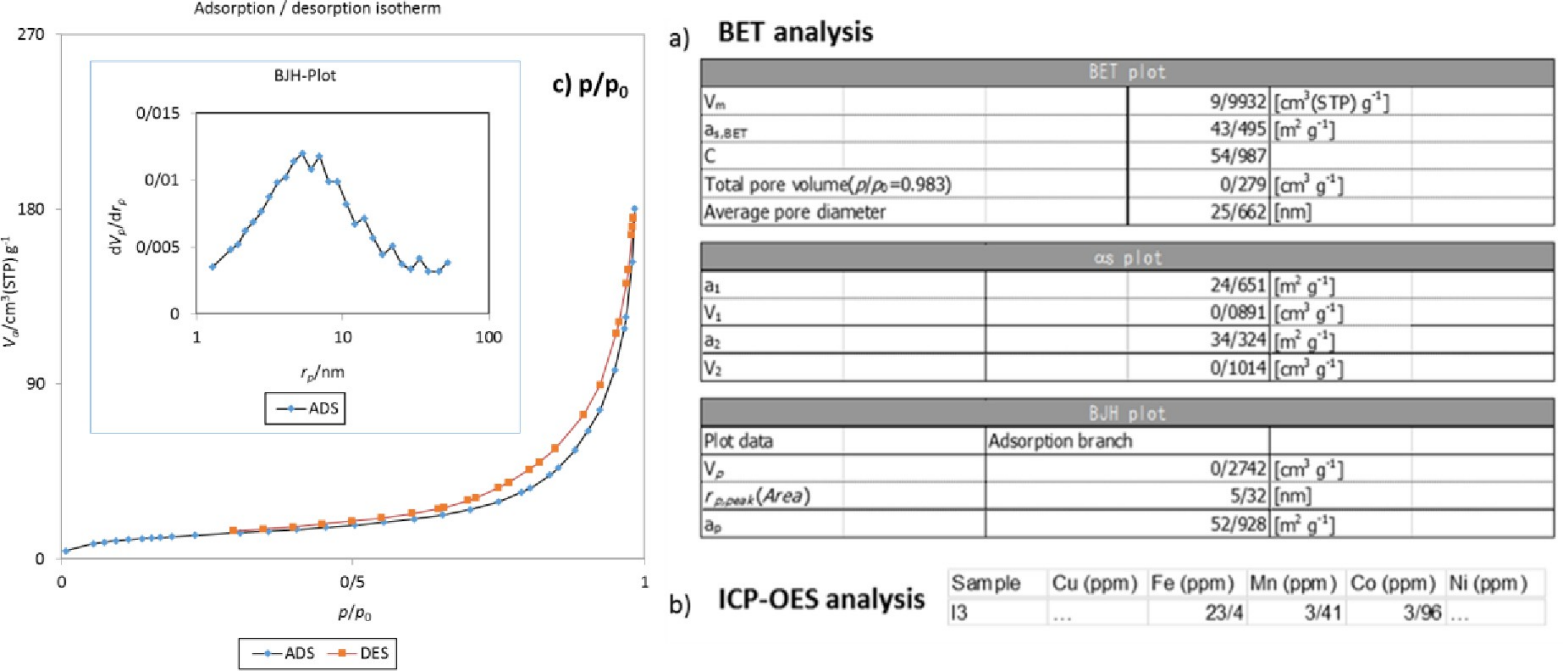
BET and ICP-OES analyses of nanocatalyst before/after of multi cross-coupling reactions. 10a, 10c) BET analysis (The size of the holes = 6–8 nm) and 10b) ICP-OES analysis.

To confirm the nature of magnetic nanocomposite, nitrogen adsorption/desorption isotherm was studied by the BET method, which identified the size distribution of the pores created in the catalyst. The isotherm (Fig 10) showed the properties of Schiff-Base aromatic rings conjugated to the magnetic nanocatalyst substrate in the range of 0.28–0.9 (P/P_0_) for cobalt/manganese bimetals (Fig 10C). In general, the aromatic ring of Lysine observed as slit-shaped pores in the plate show a large surface area with high efficiency in the magnetite nanocatalyst structure. BET surface area and pore volume of magnetic nanocomposite are approximate to 24 (a_1_ = Co)-34 (a_2_ = Mn) m^2^g^-1^ and 0.093 (a_1_ = Co)-0.1 (a_2_ = Mn) cm^3^g^-1^, respectively, are related to the cobalt and manganese bimetallic magnetic nanocatalyst, which has a large pore size (Fig 10A), the distribution of cobalt/manganese nanoparticles (25 nm), a very strong heterogeneous structure of the magnetic nanocatalyst confirmed due to the regular distribution and inclusion of cobalt and manganese nanoparticles on the morpholine substrate (organic linker), it has a greater amount of coating than palladium metal, the holes of these pores are quickly emptied after each loading and the rate of placement of organic reagents is facilitated, and A^3^-coupling reactions were repeatedly performed on the magnetic nanocatalyst substrate and the absorbed amount of organic linkers, cobalt/manganese metals compared to the unabsorbed amount was more than 98%, which confirms the coherent and heterogeneous structure of the magnetic nanocatalyst is (Fig 10).

According to the data in Fig 10B, ICP-OES analysis was used to further identify bimetals and the fixed amount of Co and Mn to be placed on the surface of Fe_3_O_4_@SiO_2_/CLM/Co/Mn magnetic nanocatalyst was 3.96 (Co) and 3.41 ppm (Mn) was evaluated. As it was previously investigated, using EDX, ICP-OES analyzes and the amount of consumed sample (g) of the nanocatalyst, the amount of millimoles of transition metals stabilized on the surface of the nanocatalyst can be measured [28]. For this purpose, according to the amount of Fe_3_O_4_@SiO_2_/CLM/Co/Mn magnetic nanocatalyst synthesized (0.1 g), along with the numbers obtained in BET analysis (Co/Mn 3.44 and 3.96 ppm, the size of the holes = 6–8 nm), respectively; 2 and 2.1 mmol of cobalt and manganese metals (II).

### Optimization of nanocatalyst

**Fig 11:**
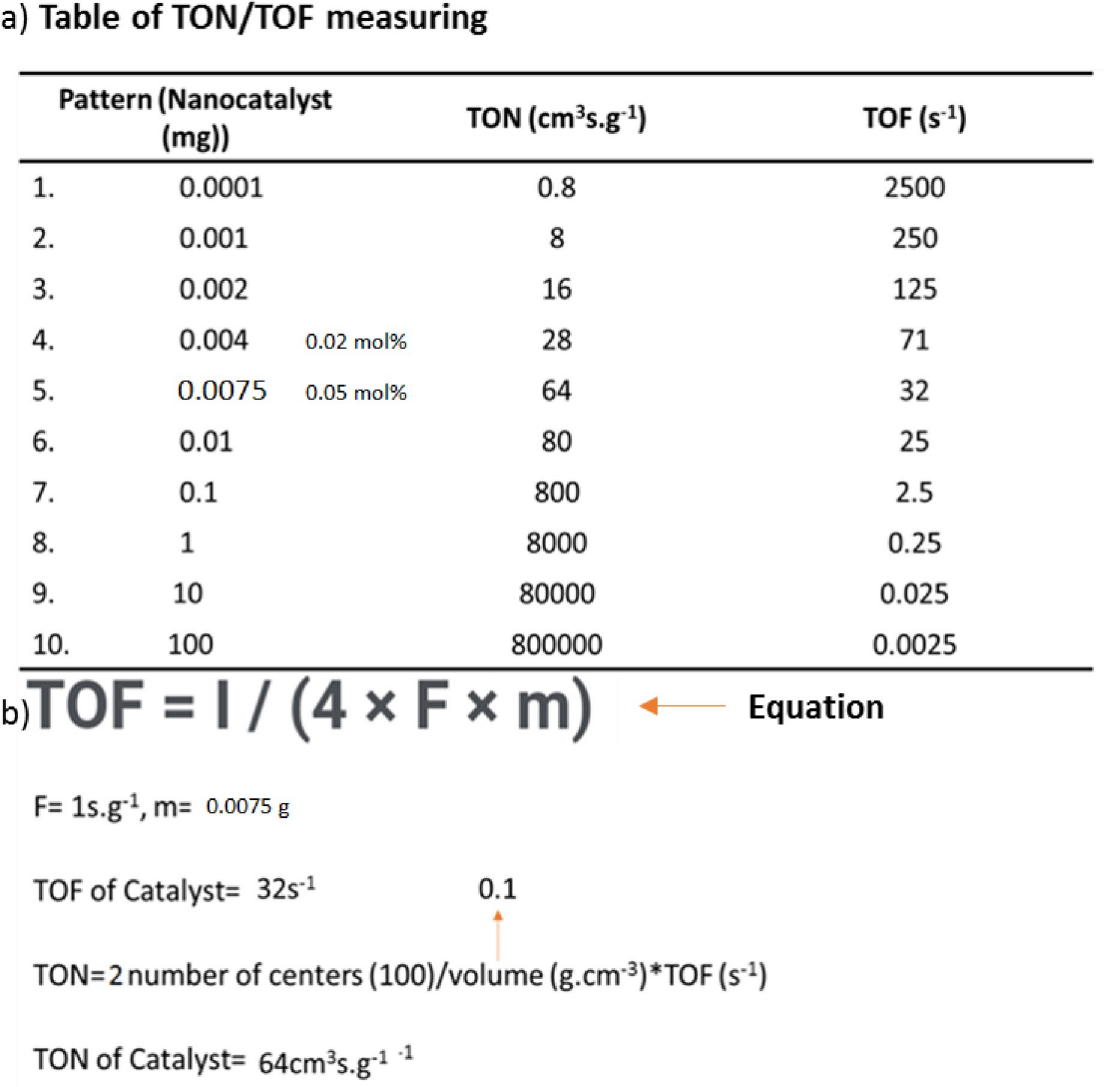
TOF and TON formula for nanocatalysts [31]. 11a) Table of TON/TOF measuring and 11b) Equation.

Based on the studies, most of the values of nanocatalysts used in coupling reactions are more than 0.1 g, which is not very favorable compared to the standard values, but the values mentioned in this project are very favorable for the use of bimetallic nanocatalysts and, according to the standard values in It is used in the laboratory and industry. The reason for the standard use of magnetic nanocomposite is to increase the efficiency of the catalyst power and prevent wastage of the sample amount, as well as to increase the percentage of the composition of the obtained products. Therefore, the amount of catalyst used in this reaction is 0.05/0.046 mol%, which is an extraordinary amount of about 0.0075 g (Fig 11A). As shown in Table 1, this amount of catalyst was sufficient to complete the reaction (Table 1, Charts 1–3). The bimetallic magnetic nanocatalyst has two capacities to perform coupling reactions, so that its heat consumption is about 15% much lower than the monometallic state, and its catalytic power is 30% higher in smaller quantities for coupling two reactants.

**Table 1:** Data^,^s for A^3^-coupling reactions.

Entry	Solvent	Amount of catalyst (mg)	Temperature (°C)	Time (min)	Yield (%) ^[^^e^^]^	TON	TOF (s^-1^)
1	CH_3_CN _(2 ml)_	Fe_3_O_4_@SiO_2_/CLM/Co/Mn (10)^c^	70	90	75	82	41
2	THF _(2 ml)_^a^	(10)	110	100	79	84	42
3	DMSO _(2 ml)_^b^	(5)	100	80	90	58	29
4	H_2_O _(2 ml)_	(5)	70	70	82	62	31
5	Dioxane _(0.2 ml)_	(0.5)	25	60	80	84	42
6	Dioxane _(0.5 ml)_	(1)	35	55	84	75	37.5
7	Dioxane _(5 ml)_	(2)	50	45	90	72	36
8	Dioxane _(5 ml)_	(4)	68	35	92	63	30.9
**9**	Dioxane _**(5 ml)**_	**(7.5)**	**68**	**30**	**98**	**57.14**	**12.5**
10	Dioxane _(10 ml)_	Fe_3_O_4_ (20)	100	22h	trace	87	43.5
11	Dioxane _(10 ml)_	Fe_3_O_4_@SiO_2_/CLM (20)^d^	100	20h	20	83	41.5
12	Dioxane _(10 ml)_	Co/Mn (OAc)_2_ (20)	70	14h	65	72	36
13	Dioxane _(10 ml)_	Fe_3_O_4_@SiO_2_/CLM/Co(7.5)	68	1.5h	87	68	34
14	Dioxane _(10 ml)_	Fe_3_O_4_@SiO_2_/CLM/Mn(7.5)	68	2h	82	78	39

^a^ Tetrahydrofuran

^b^ Dimethyl Solfoxide

^c^ Fe_3_O_4_@SiO_2_/3-Chloroiso-propyltriethoxysilane-Lysine-Morpholine/Cobalt (II)/Manganese (II)

^d^ Fe_3_O_4_@SiO_2_/3-Chloroiso-propyltriethoxysilane-Lysine-Morpholine

^e^ Yield refer isolated products

Also, according to Table 1, the results of the data show that the monometallic magnetic nanocatalysts made in rows 13 and 14 have an efficiency of 82–87% compared to row 9 in the timing above 1 h. As a result, the monometallic manganese and cobalt magnetic nanocatalysts have achieved a higher time and lower efficiency than the bimetallic manganese/cobalt magnetic nanocatalyst, which confirms the high quality and efficiency of bimetallic to monometallic.

### Solvent forms

**Fig 12:**
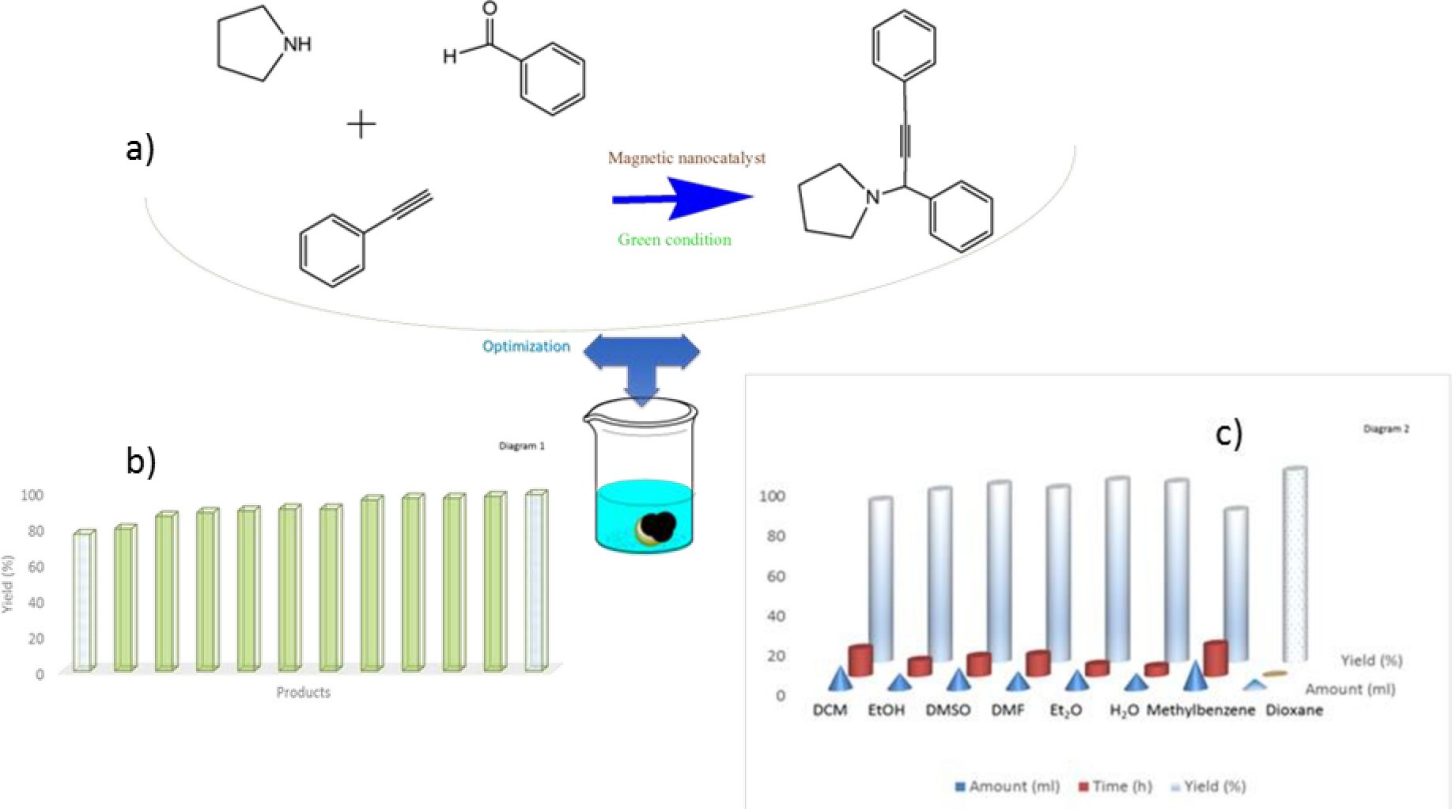
A^3^-coupling reactions with optimism of reaction conditions in diagrams. 12a) general reaction of A^3^ coupling, 12b) Diagram 1 (products of A^3^ coupling) and 12c) Diagram 2 (optimum of solvents and times).

Sometimes a series of reactions does not need a solvent, but it is almost impossible to do a reaction without using a solvent, or if it is, it is not economical. The use of solvent to create a suitable atmosphere in the effective collision of two reactants in completely ideal conditions (lower reaction temperature, less use of suitable bases and salts) so that the energy consumed by the ionizing solvent is recovered at the end of the reaction. Therefore, the role of the solvent is very effective in bringing the two phases closer between the two reactants through the solvent coating and providing the exchange of functional groups and the creation of a covalent bond between the two organic reactants. Because there are three different pair reactions, a strong base is needed to help remove hydrogen from both sides of the reactants, so that by dehydrogenating it, a compound with a new covalent bond is formed as a pair (to perform A^3^-coupling reactions). The solvent used varies depending on the coupling reaction, but in general the solvent used should be green, which is dioxane with amount of optimum about 5ml (Table 1, Fig 12C, Diagram 2).

### Timing processing

One of the important parameters that helped the reactants to be activated and sit on the magnetic nanoparticles is the presence of the right temperature and giving enough time for their loading, as well as the interaction between the reactants (Table 1, Fig 12C, Diagram 2). The temperature should be ideal and should not be too high because it will cause some of the reactants to evaporate and be destroyed before the reaction, so the ideal temperature here is 70°C. One of the main reasons for using bimetallic magnetic nanocatalysts here is to reduce the reaction time. According to the data in Table 1, the reaction time is generally in the range of 15–60 min compared to the values in Table 3, for the other nanocatalysts (all of which took more than 60 min to complete the coupling reaction), where this timing is completely standard and optimal.

### A^3^-coupling reactions

**Fig 13:**
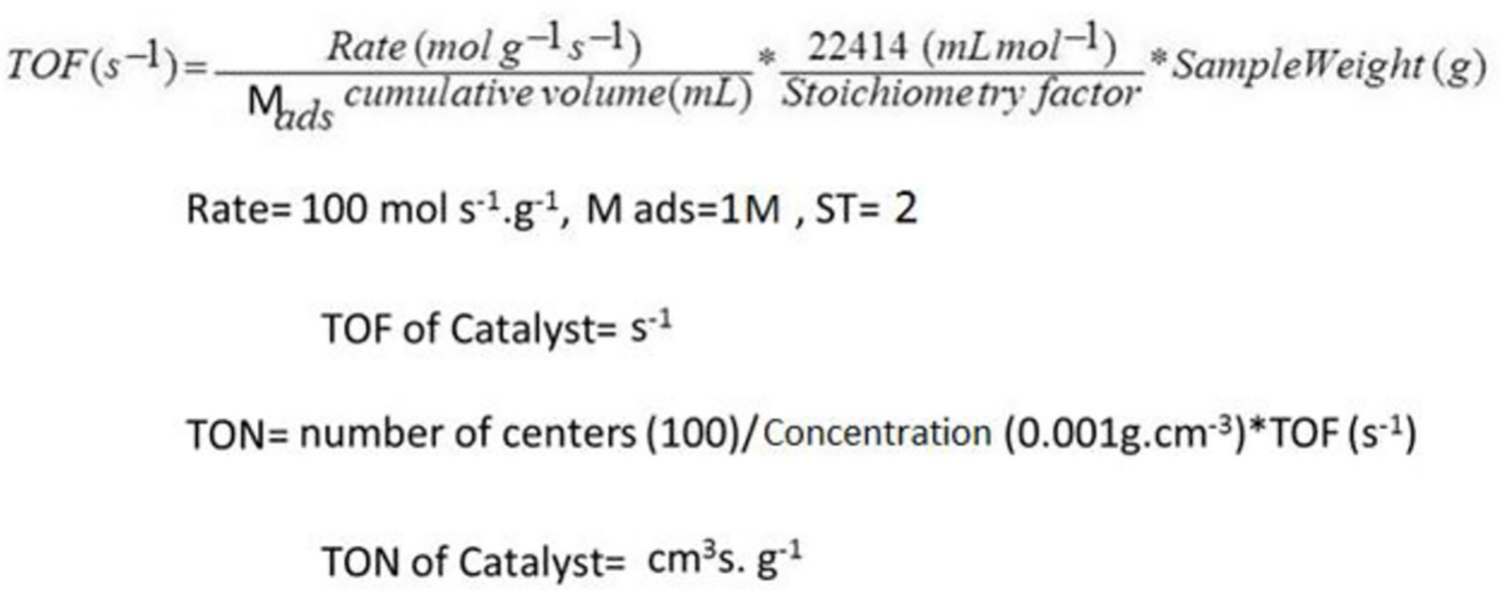
TON/TOF measuring of multi cross-coupling products [37]. TON/TOFs values for evaluating A^3^-coupling reaction products.

Second, unique nanocatalyst has a high reactivity that depends on several factors, one of which is that it is magnetically controlled by an external magnetic field, which helps to improve the reaction process and is easily separated from the reaction medium by the same field. Another factor is the presence of two strong metals, Co and Mn, with a capacity of (II to V), which are coated on the surface of the nanocatalyst and greatly increase the catalytic power of the nanocatalyst and so TON/TOF is measured in Fig 11B, comparing of TON/TOF magnetic nanocatalyst in A^3^-coupling reactions is made in Table 2. Based on the data in Table 2, the TON/TOF measured values show that the magnetic nanocatalyst did not change much during the A^3^-coupling reaction (Fig 13). Based on this, it is concluded that the cobalt/manganese bimetallic magnetic nanocatalyst has a heterogeneous surface and a stable crystal structure. This A^3^ coupling reaction causes the synthesis of a new chiral compound in the final composition of the reaction product, which has been synthesized for the first time (Fig 12A and 12B). This reaction is actually a reaction between compounds of benzaldehyde (electrophilic position) and a phenyl acetylene, pyrrolidine (nucleophilic position) at 70°C, and the solvent is dioxane and measuring of TON/TOF is brought for nanocatalyst (Fig 13). The obtained products have a high efficiency of 76–98% and a selectivity of 99%. In the following, the general conditions of the reaction have been carefully examined (Fig 12B).

**Table 2:** Comparing of TON/TOF magnetic nanocatalyst in A^3^-coupling reactions.

Entry	Agent groups	TON (s^-1^)	TOF (cm^3^s.g^-1^)
1. m = 0.0208	-OH	58.75	11.99
2. m = 0.02079	-NH_2_	59.52	12.02
3. m = 0.0223	-NO_2_	63.69	11.22
4. m = 0.0217	2-OH	62	11.52
5. m = 0.0224	-OH, -OCH_3_	63.98	11.17
6. m = 0.0231	2-OCH_3_	65.96	10.83
7. m = 0.0216	2-NH_2_	61.69	11.58
8. m = 0.02228	2-NO_2_	63.73	11.21
9. m = 0.0215	2-CH_3_	61.42	11.63

### Original studies

**Fig 14:**
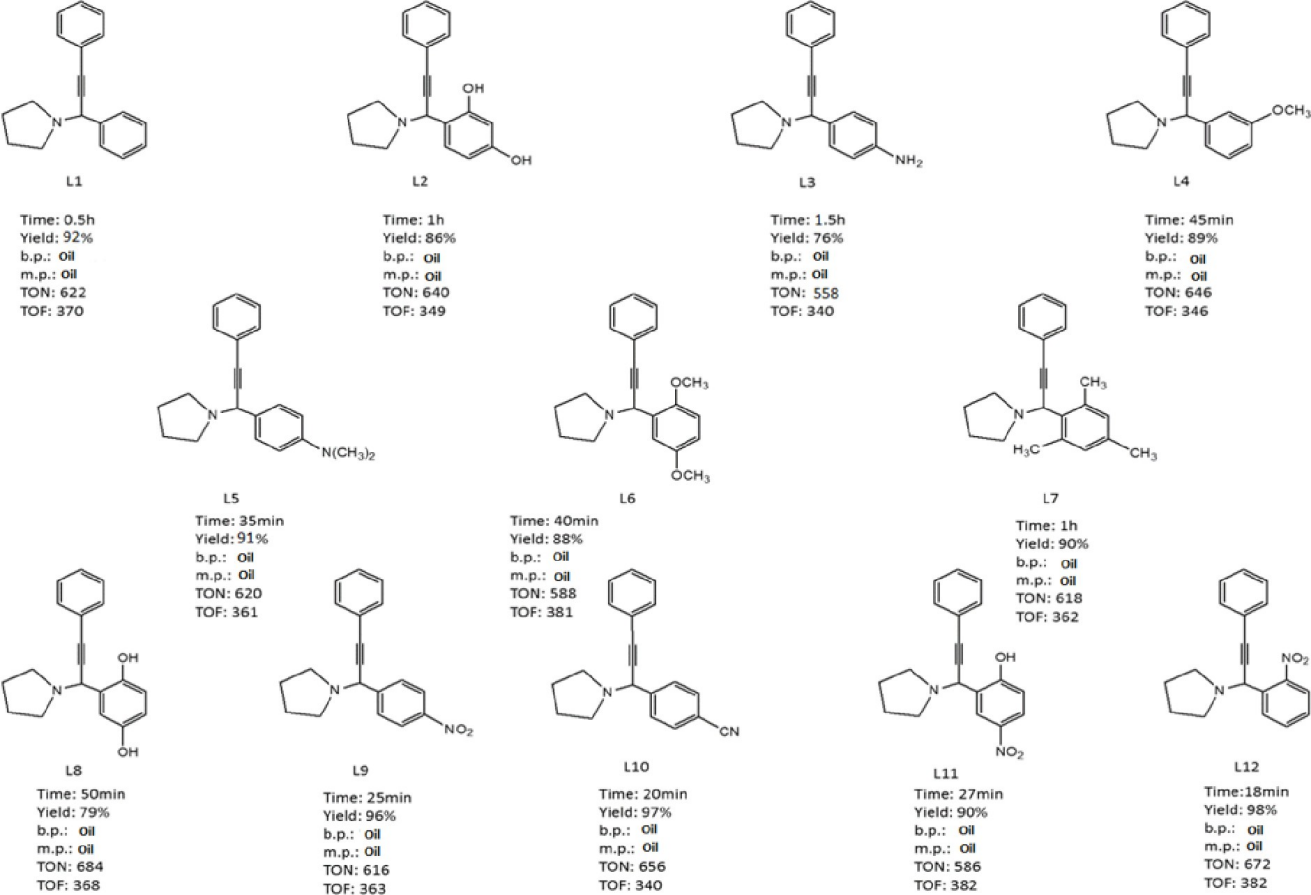
Standardized of A^3^-coupling products by melting point. Times, yields % and TON/TOFs of products (L1-L12) which is the least yield for L3 and the most for L12 products.

**Fig 15:**
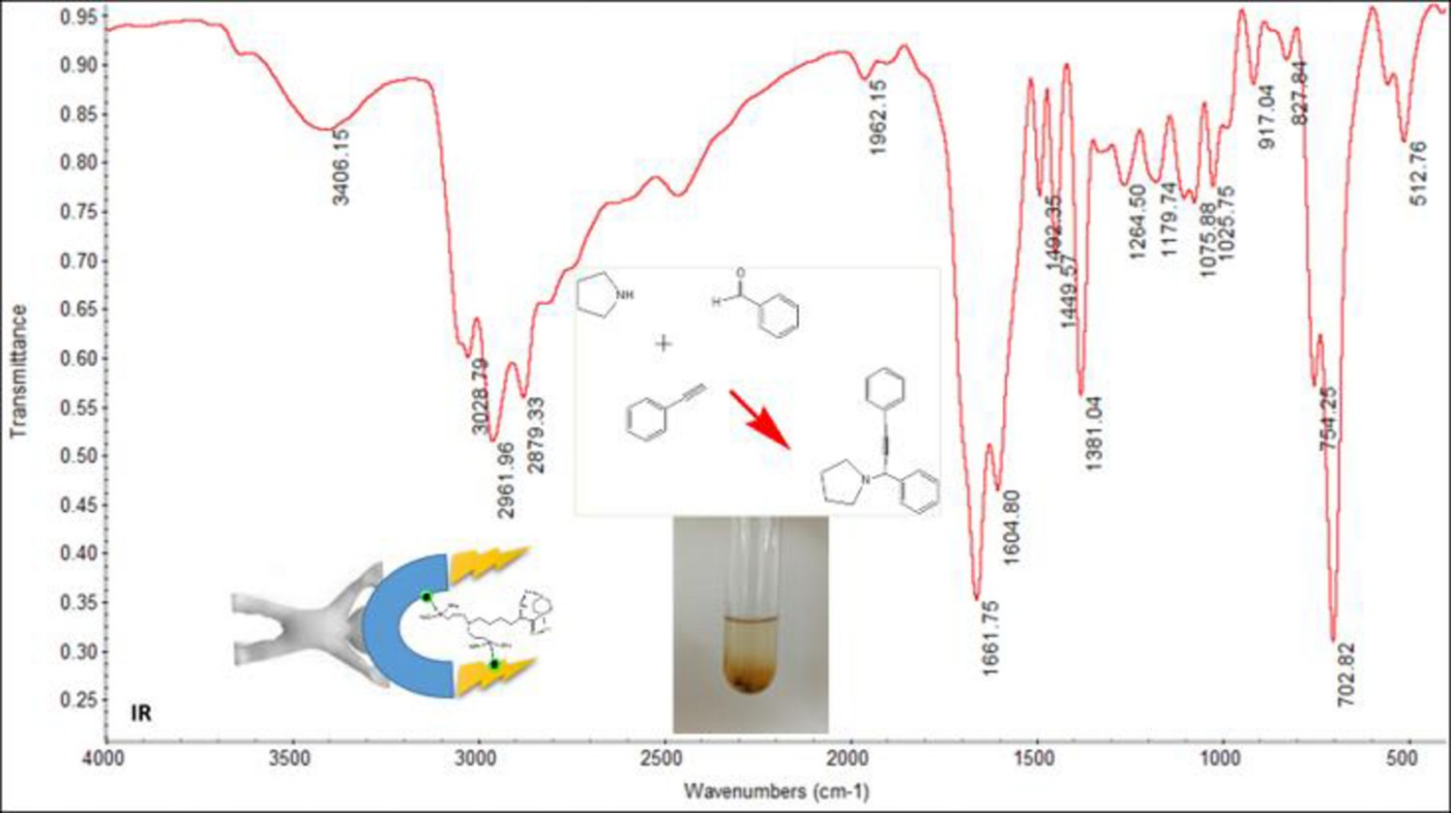
IR analysis of A^3^-coupling product. This analysis is used for the detection of 1-(1,3-diphenylprop-2-yn-1-yl)pyrrolidine functional groups.

In order to identify the products, during the reaction, the TLC solution is taken after 5 minutes to apply a stain of the reaction solution on the TLC paper made, in the next stains are applied to the two detectors. The initial reactants and finally, in a special reservoir of hexane-ethanol-ethyl acetate solvents are purified, in certain proportions (non-polar to polar). We must first observe 3 stains unless a reagent is completely used in the reaction, in which case we must wait until the next stain is removed (ie the reaction time is complete). Finally, the solvent should be allowed to leave the surface of the TLC paper and the resulting stains to be seen under the UV lamp. In this case, where the product composition percentage is 100%, the organic product is easily separated from the aqueous phase by an organic solvent by the decanter funnel (eg ethyl acetate), but if another stain is seen next to the product stain, a column should be used that was made of silicate and the main stain removed from the solution and finally filtered to obtain a melting point if it was not liquid at room temperature. A table containing sufficient information on the reaction conditions of A^3^-cyclic couplings with each of the reactants (percentage of product obtained above 98%, melting point of the products (L1-L12) with an accuracy of close to 99% with predicted actual values) depending on the time, their melting point data`s is brought (Fig 14). By examining the reaction conditions with other catalysts in Table 1, it can be concluded that the current nanocatalyst (last row) has ideal conditions compared to other catalysts. Finally, for further studies, a tablet with potassium bromide should be prepared from its powder and analyzed on an IR device for 1-(1,3-Diphenylprop-2-yn-1-yl)pyrrolidine (Fig 15).

The basic reasons that make the magnetic nanocatalyst in this project preferred over other nanocatalysts (in Table 3) are very clear. The general characteristic of the nanocatalyst is that it can be controlled with an external field due to its magnetic properties and it can be separated by approximately 99% without causing side effects in the coupling reaction. Its structure consists of a silica coating and contains 3-chloroisopropyl triethoxysilane, Lysine, morpholine ligands and also has two very powerful metals, cobalt and manganese, which is a very unique and complete structure that enables the coupling reactions of A^3^-coupling with the ability it achieves over 96%, which is more than 35% more efficient compared to other nanocatalysts. The amount of the sample used is about 0.001 times less, which is very ideal, so that it is sufficient for the coupling reaction to produce products with a high percentage on an industrial scale in the amount of kilograms and tons, around grams. Regarding the timing needed to perform each of the coupling reactions, less than one hour was required, which is also very ideal. The solvent used is a harmless and green solvent (i.e. ethanol) which, in addition to creating green products, is at a lower temperature than other catalysts, which can be seen in Table 3. Finally, it was used again on a smaller scale (around 0.02 mol% of Fe_3_O_4_@SiO_2_/CLM/Co/Mn) with a high efficiency of 99% and also the obtained products have a combination percentage of 92% and a selectivity of 99% (Fig 14, Diagram 1). By summarizing these things, it is concluded that the magnetic nanocatalyst Fe_3_O_4_@SiO_2_/CLM/Co/Mn is very special, widely used and more powerful than other nanocatalysts.

**Table 3:** Comparing of Fe_3_O_4_@SiO_2_/CLM/Co/Mn nanocatalyst to other catalysts.

Entry	Catalyst	Conditions	Time (h)	Yield (%)^[^^g^^]^	References
1	Ni(II)/Fe (0.2 mol% Ni)^a^	H_2_O/90° C	6	94	[44]
2	ZnFe (0.524 mol% Zn)^b^	Hexamine/H_2_O/Reflux	10	85	[45]
3	FeSi (0.5 mol% Pd)^c^	DMF/120° C ^e^	1.25	93	[46]
4	NHC-palladium complex (1 mol% Pd)^d^	DMSO/100°C ^f^	1	81	[49]
5	Fe_3_O_4_@SiO_2_/Schiff base/Pd(II) (0.5 mol% Pd)	DMF/90° C ^e^	1	93	[50]
6	Nano Cu@Fe_3_O_4_ (1 mol% Pd)	DMF/110°C ^f^	24	83	[51]
7	[CuCl(SeCH_2_CH_2_CH_2_NMe_2_)]_2_ (3 mol% Pd)	DMF/100° C	12	92	[52]
8	Fe_3_O_4_@SiO_2_/CLM/Co/Mn (0.05/0.046 mol%)^d^	Dioxane/70-101° C	18-60min	>96	present

^a^ Nickel/Fe_3_O_4_

^b^ ZnO/Fe_3_O_4_

^c^ Fe_3_O_4_@SiO_2_-palladium

^d^ Fe_3_O_4_@SiO_2_/3-Chloroiso-propyltriethoxysilane

^e^ Dimethylformamide

^f^ Dimethylsulfoxide

^g^ Yield refer isolated products

### Chirality of propargylic compounds

Measurement of chirality of propargylic compounds (A^3^) is done by polarimetric analysis. Before checking the degree of chirality of the products, an important matter must be evaluated first. With a brief look at the structure of Fe_3_O_4_@SiO_2_/CLM/Co/Mn magnetic nanocatalyst, the presence of lysine organic linker has caused the nanocatalyst to have chiral properties. This nanocatalyst gives special chirality to propargylic products in reaction A^3^ and leads to the creation of single chiral products with special chiral selectivity. All rotation values shown are from these compounds with the addition of optical purity calculated as R % or S % yield. Considering that the synthesized catalyst has a lysine bond on its substrate, it has special chirality for controlling and purifying propargylic compounds. According to previous researches [31–39], the value of specific optical rotation, enantiohexene (selectivity or measurement of excess enantiomer) and optical purity for the compound C_19_H_19_N (1-(1,3-Diphenylprop-2-yn-1-yl)pyrrolidine) is equal to +30.5°, %ee = 85% and o.p. = 98% obtained using the formula [α] = α/l.c. In this research, in order to calculate the chirality of the compound C_19_H_19_N under the conditions (temperature 20°C, wavelength 589 nm of sodium lamp and 10 dm sol), using the magnetic nanocatalyst Fe_3_O_4_@SiO_2_/CLM/Co/Mn, specific optical rotation = +26° (with a coefficient of 0.01), %ee = 93% and o.p. = 85% were obtained. The comparison of the show results confirms that Fe_3_O_4_@SiO_2_/CLM/Co/Mn nanocatalyst has a high efficiency in optical purity and enantioxenia, and this efficiency is 25% higher than other nanocatalysts [38].

**Fig 16:**
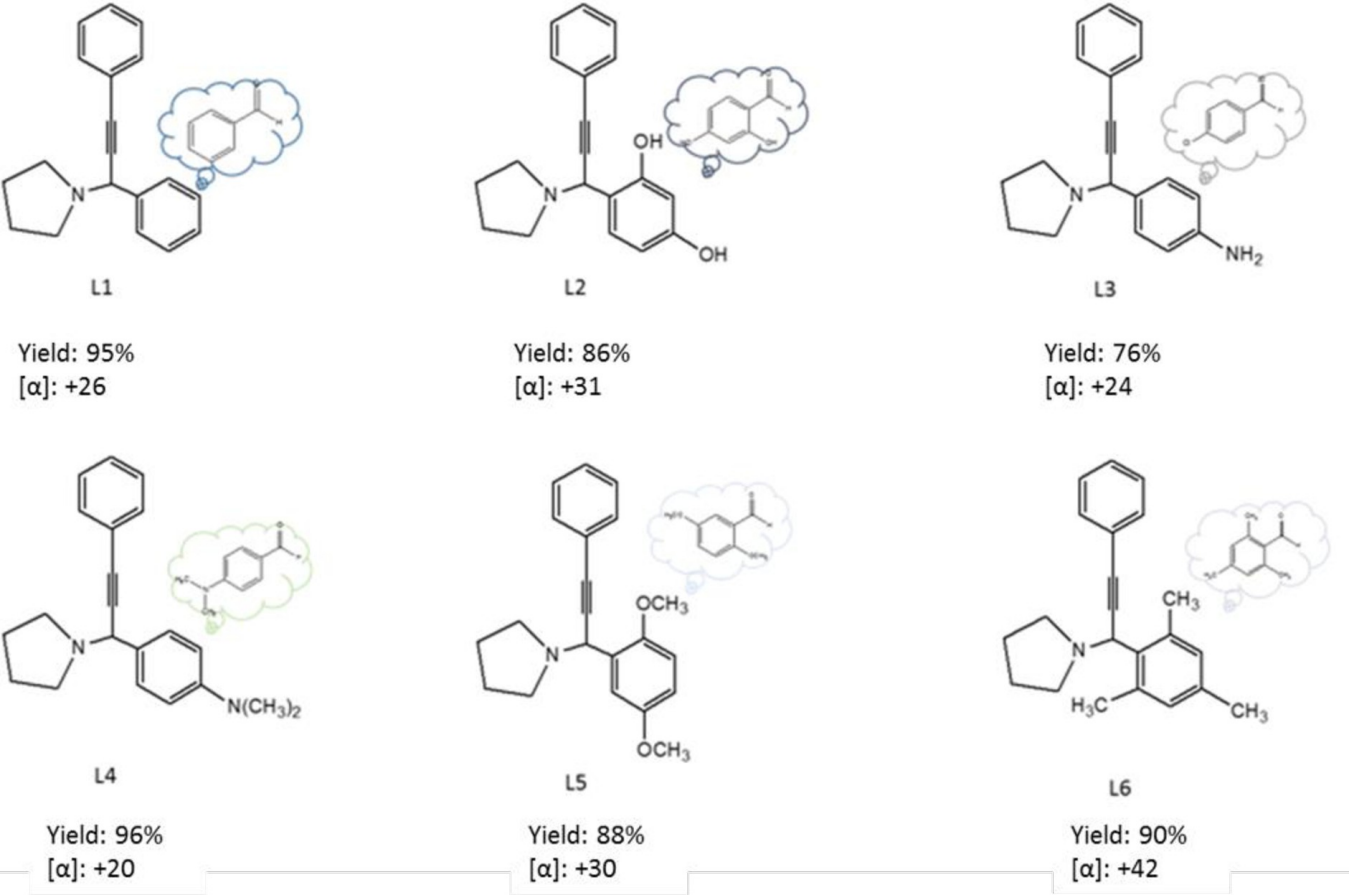
Optical rotation of A^3^-coupling products with study on [31–39]. For L1-L6 products, the amount of optical rotation is evaluated as positive.

Sue to Fig 16, measuring chirality of propargylic compounds (L1-L6) is showed by polarimetry analysis. All amounts are a showed rotation of these compounds by addition an optical purity that is calculated to earning of R % or S %. Due to the fact that the synthesized catalyst has a lysine linker on its substrate, it has a special chirality to control and purify propargylic compounds by special optical rotation [α]. So that from the values visible in Fig 16, the effectiveness of catalytic chirality is above 60%, which causes positive enantiomeric purity in all A^3^-coupling derivatives.

### Impact of Schiff’s base nanocatalyst

As previously discussed, due to the presence of a Lysine ligand in the nanocatalyst structure, which is an alpha amino acid structure [57], its acidic carboxylic group cannot bond with the morpholine ligand due to strong resonance. For this purpose, as shown in Fig 2, a tosyl compound (1 mmol) was used, which is easily placed in place of the hydroxyl group (hydroxyl from carboxylic acid) and the tosyl position is a very suitable leaving group. Finally, the acidic pH is reduced in this way and a covalent amide bond is formed between the morpholine nitrogen and the ketone group of Lysine (with tosyl). So, here the acidic site is attacked by the nitrogen position of the morpholine ligand and creates an open Schiff-base of these amide bonds are very important in the formation of chemical and biological compounds. This pH is close to 12 because the acidic strength decreases with the formation of amide bonds, and it becomes more difficult for metals to occupy nitrogen-oxygen positions due to resonance, and they are more likely to be on the side of free nitrogen. The result was that a suitable substrate was created by Schiff-bases for loading cobalt/manganese metals on the surface of the magnetic nanocatalyst and increasing the surface efficiency for A^3^-coupling reactions up to 20%.

### Coupling reaction process

**Fig 17:**
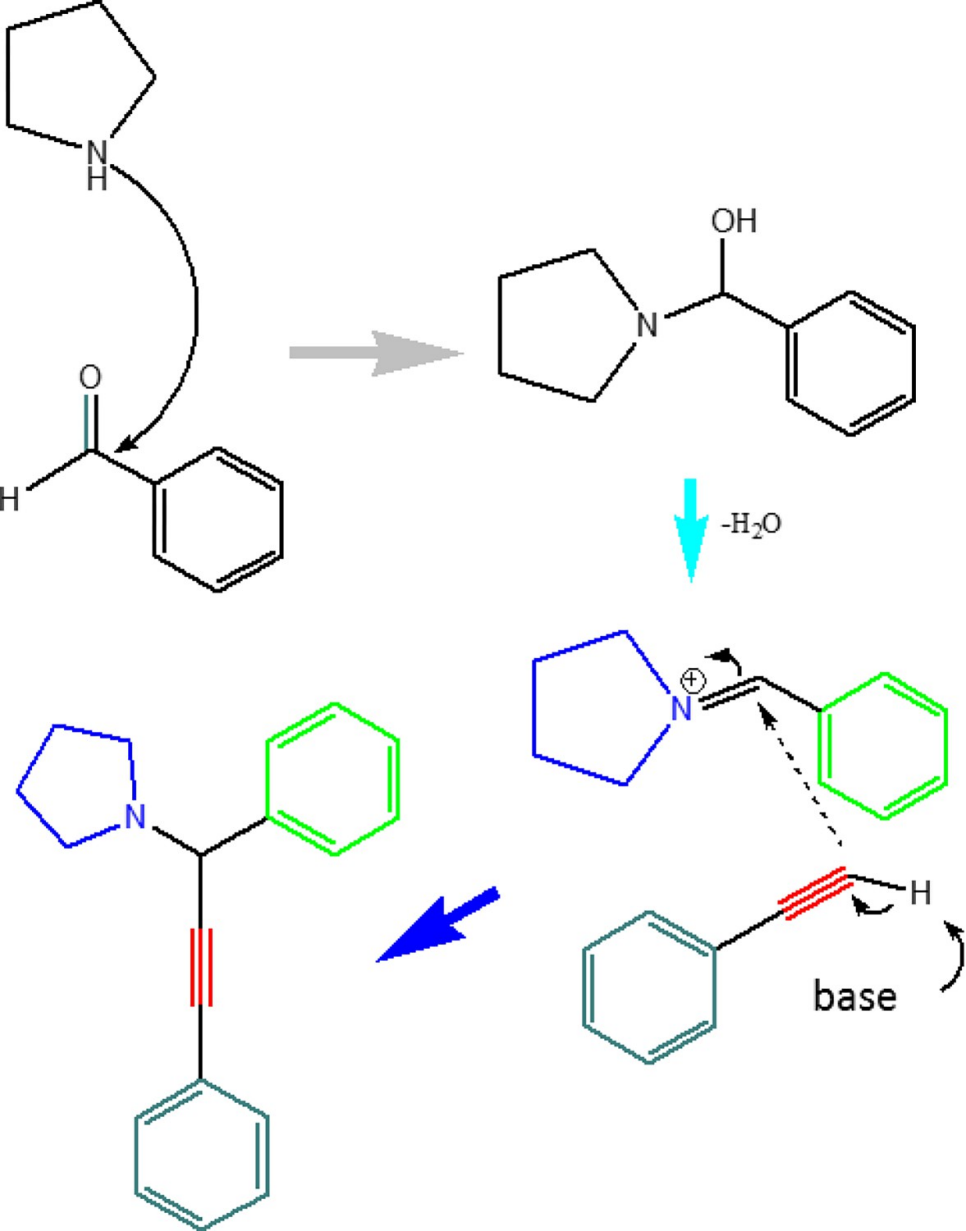
Full reactions of A^3^-coupling. The mechanism of 1-(1,3-diphenylprop-2-yn-1-yl)pyrrolidine product formation is generally shown in the presence of a base.

The basis of this work is actually a three-step reaction, in which bimetallic magnetic nanocatalyst must be used for each of the couplings. So that (first) the aldehyde carbon group of benzaldehyde is attacked with a nitrogen group of morpholine compound. Next, an imide bonding is made (of interaction between two electrophilic/nuleophilic centers) to in finish, a water group is extracted of reaction and a enamide group is be obtained. In the third step, the phenyl acetylene compound is inserted into the N-amide compound by a general base, and at this moment the third compound (i.e. propargylic) is made by A^3^-coupling reaction. Finally, the desired compound is a chiral and that should be measured by polarimetry analysis (Fig 17). The products of this reaction are obtained by A^3^-coupling reaction, and as can be seen in Fig 14, can be used in the manufacture of biological and natural structures that have useful medicinal and therapeutic properties.

### Mechanism, recovery and reuse of nanocatalysts

**Fig 18:**
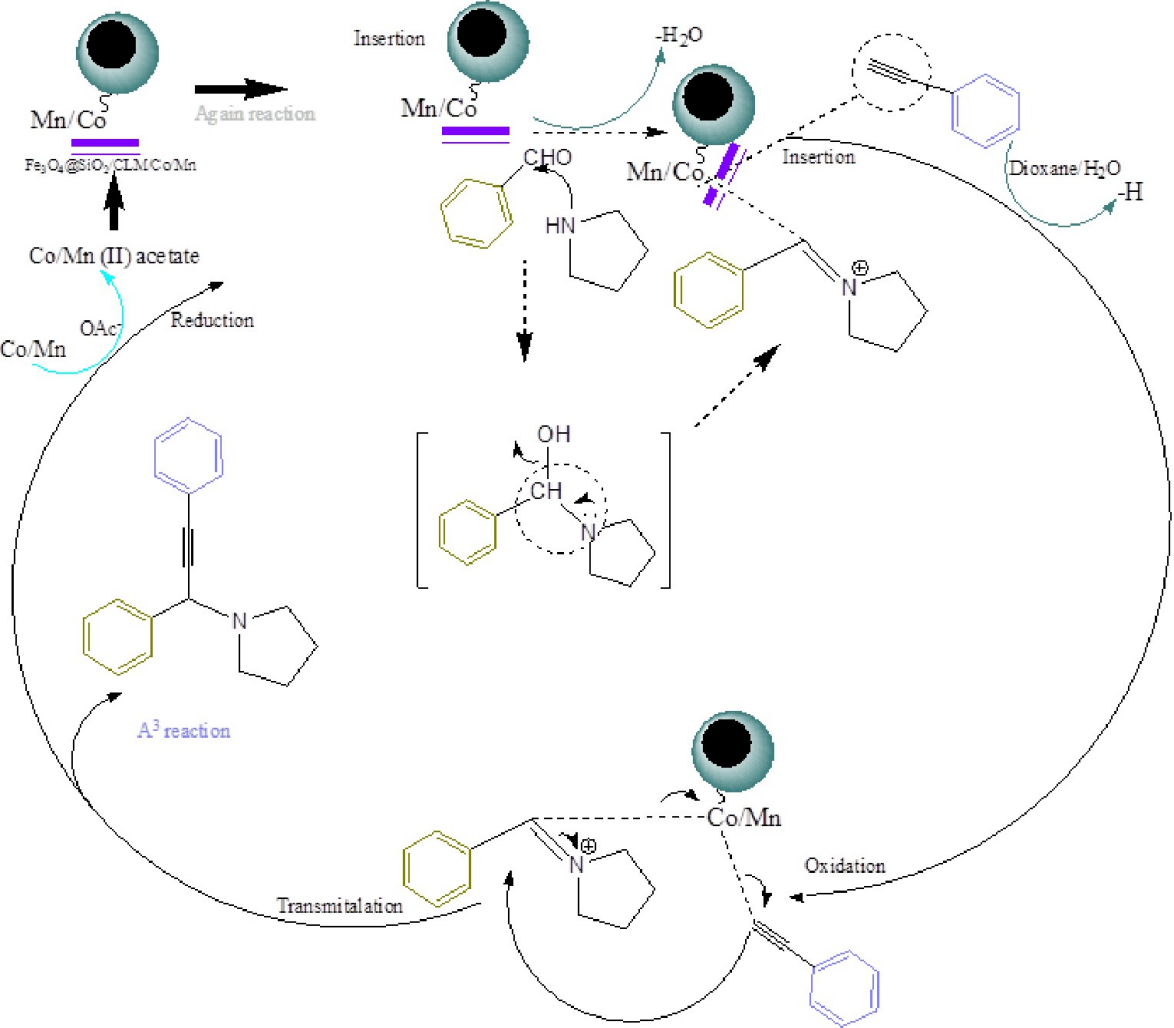
Mechanism of A^3^-coupling reaction. The multicomponent synthesis reaction was carried out in the presence of Fe_3_O_4_@SiO_2_/CLM/Co/Mn bimetallic magnetic nanocatalyst, and the proposed mechanism for it was presented.

Finally, a proposed mechanism is presented that shows the overall reaction process (Fig 18). This mechanism is one of the most recent mechanisms that have a new design by new nanocatalyst. The cobalt/manganese bimetallic magnetic nanocatalyst is in the form of cobalt II/manganese II (Co (II)/Mn (II)) in the basic state, which are first converted into same form, during the insertion stage to perform the A^3^-cyclization reaction. In this step, two Co (II)/Mn (II) metals are connected to two molecules of benzaldehyde and pyrrolidine, and since the reaction is carried out on two metals (bimetallic using). In the next step, when the benzaldehyde compound (aldol group is changed to alcohol group, reduction processing) must be changed to hydrous compound by nitrogen group of morpholine. Finally, with attacking of phenyl acetylene compound to N-amide synthesis in presenting of a general base, the cobalt/manganese metals are extracted by magnetic field and A^3^-coupling product is made.

**Fig 19:**
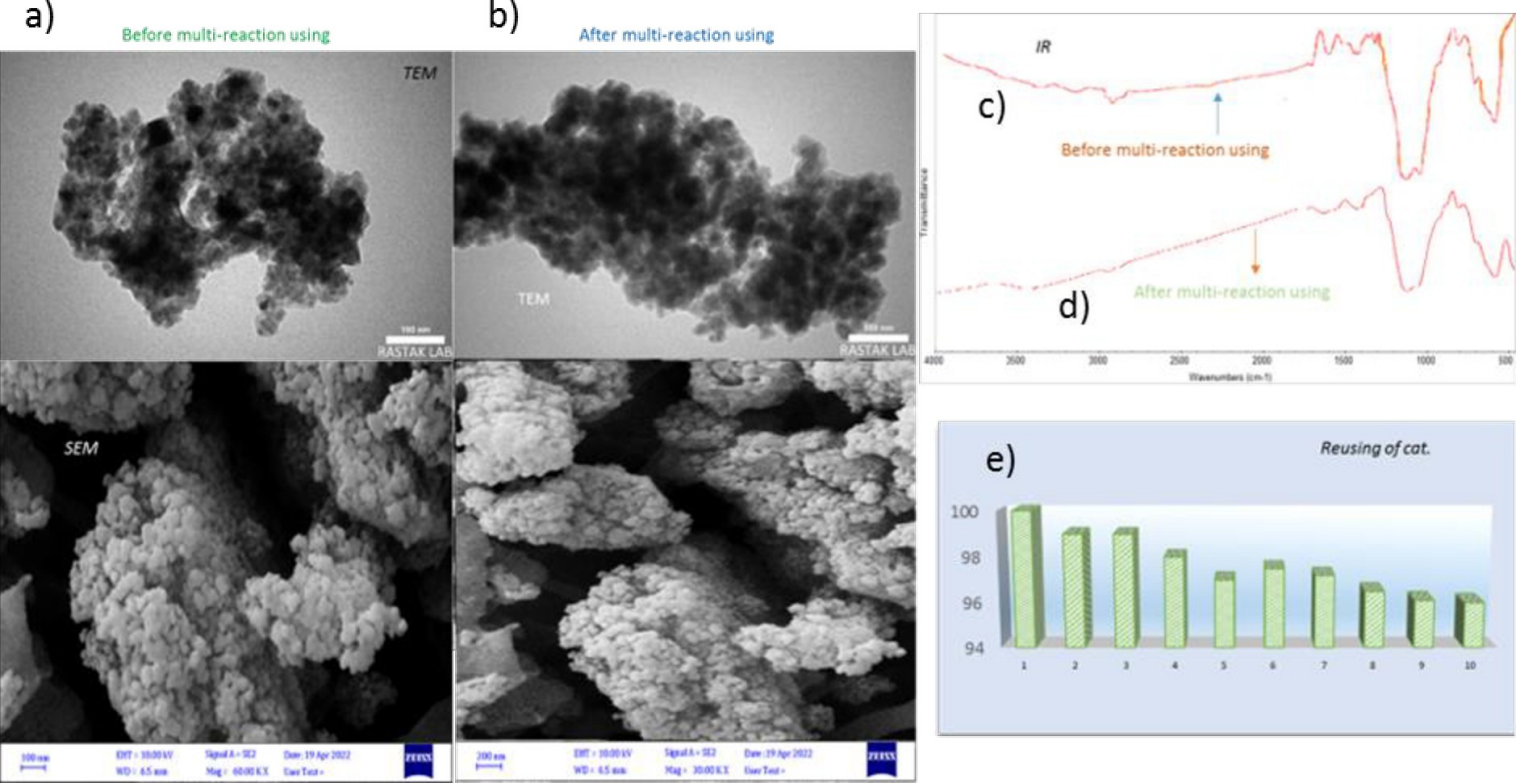
IR, SEM and TEM analyses with diagram of 10 periods of Fe_3_O_4_@SiO_2_/CLM/Co/Mn nanoparticles recycling reuse. 19a) Before multi-reaction reusing, 19b) After multi-reaction reusing, 19c) IR analysis of before multi-reaction reusing, 19d) IR analysis of after multi-reaction reusing and 19e) Reusing chart of Fe_3_O_4_@SiO_2_/CLM/Co/Mn magnetic nanocatalyst.

At the end of the work, the strength of the catalyst should be proven, so that after the first reaction, the nanocatalyst is washed and dried several times and tested in a new reaction to evaluate its performance. According to the graph data, the current nanocatalyst has nearly 96% capacity, and after about 10 reuses, only 4% capacity is reduced, which can be analyzed by SEM, TEM (Fig 19A and 19B), and IR analyses (Fig 19C and 19D). The data in Fig 19E show that no structural or functional quality of the catalyst is reduced and the nanocatalyst performs the A^3^-coupling reactions very powerfully.

### Leaching

**Fig 20:**
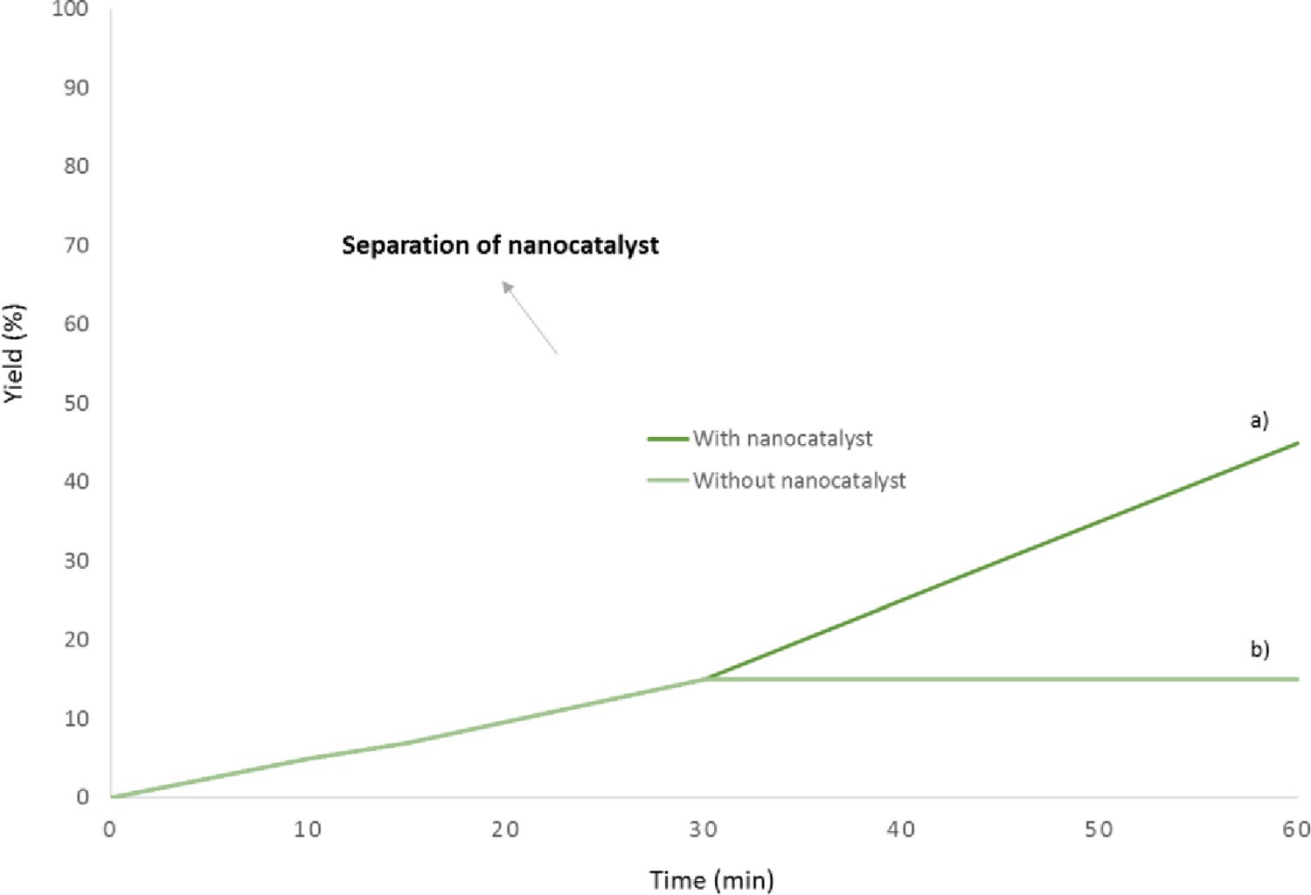
Leaching analysis of nanocatalyst before and after reaction. 20a) Leaching measuring with nanocatlyst in full time that the slope of the graph was increasing and 20b) Leaching measuring without nanocatlyst in half the reaction completion time that the reaction was stopped.

Leaching is an important factor in measuring the catalytic rate of nanocatalysts with coated metal on its surface, which is used here to investigate the rate of leaching of two metals, cobalt and manganese, on the surface of magnetic nanocatalysts (homogeneous presence or heterogeneity of the catalyst), was evaluated based on the filtration test. The reaction was stopped after 27 minutes (half of the reaction time) and the catalyst was separated from the reaction medium by the external field and the reaction vessel continued again (Fig 20B). However, the solution without catalyst after 55 minutes (which was checked every moment by TLC) did not see any progress in the reaction process, which confirmed the inhomogeneousness of the magnetic nanocatalyst (Fig 20A).

## Conclusion

Our goal in this project is to create new and very important couplings (A^3^-couplings) that can help a lot to make many natural and synthetic compounds in the laboratory from carbon and nitrogen couplings. What makes this project even more special is the use of a magnetic nanocatalyst that can be controlled by a magnetic field, easily accelerates the reaction between the reactants, and is easily separated from the reaction medium. The obtained products have an efficiency of more than 98% and contain a new propargylic compounds. The catalyst has used more than 96% of its power to perform these reactions without reducing the efficiency. With the scientific progress in the future world, there is a space to develop such synthetic compounds with a unique catalyst like this catalyst (Fe_3_O_4_@SiO_2_/CLM/Co/Mn). By looking at the synthesized compounds, the importance of creating such couplings becomes more important for the high synthetic and therapeutic applications in the chemical and medical industries.

## Abbreviations

MNPs: Magnetic nanoparticles
CLM: CPTES/Lysine/Morpholine
FE-SEM: Field emission scanning electron microscopy
TEM: Transmission electron microscopy
VSM: Value stream mapping
ICP: Inductively Coupled Plasma) Spectroscopy Brunauer-Emmett-Teller (BET
FTIR: Fourier Transform Infrared Spectroscopy
EDX: Energy-dispersive X-ray spectroscopy
TGA: Thermogravimetric analysis
XRD: X-ray diffraction
UV: Ultraviolet–visible spectroscopy
VSM: Vibrating-sample magnetometer
